# The ATG5 interactome links clathrin-mediated vesicular trafficking with the autophagosome assembly machinery

**DOI:** 10.1080/27694127.2022.2042054

**Published:** 2022-04-07

**Authors:** Kiren Baines, Kazuaki Yoshioka, Yoh Takuwa, Jon D. Lane

**Affiliations:** 1 Cell Biology Laboratories, School of Biochemistry, University of Bristol, University Walk, Bristol, BS81TD, UK; 2 Department of Physiology, Kanazawa University Graduate School of Medical Sciences, 13-1 Takara-machi, Kanazawa Ishikawa 920-8640, Japan

**Keywords:** ATG5, ATG12, autophagy, clathrin, endocytosis, HIP1R, PIK3C2A, proteomics

## Abstract

Autophagosome formation involves the sequential actions of conserved ATG proteins to coordinate the lipidation of the ubiquitin-like modifier Atg8-family proteins at the nascent phagophore membrane. Although the molecular steps driving this process are well understood, the source of membranes for the expanding phagophore and their mode of delivery are only now beginning to be revealed. Here, we have used quantitative SILAC-based proteomics to identify proteins that associate with the ATG12–ATG5 conjugate, a crucial player during Atg8-family protein lipidation. Our datasets reveal a strong enrichment of regulators of clathrin-mediated vesicular trafficking, including clathrin heavy and light chains, and several clathrin adaptors. Also identified were PIK3C2A (a phosphoinositide 3-kinase involved in clathrin-mediated endocytosis) and HIP1R (a component of clathrin vesicles), and the absence of either of these proteins alters autophagic flux in cell-based starvation assays. To determine whether the ATG12–ATG5 conjugate reciprocally influences trafficking within the endocytic compartment, we captured the cell surface proteomes of autophagy-competent and autophagy-incompetent mouse embryonic fibroblasts under fed and starved conditions. We report changes in the relative proportions of individual cell surface proteins and show that cell surface levels of the SLC7A5-SLC3A2 amino acid transporter are influenced by autophagy capability. Our data provide evidence for direct regulatory coupling between the ATG12–ATG5 conjugate and the clathrin membrane trafficking system and suggest candidate membrane proteins whose trafficking within the cell may be modulated by the autophagy machinery.

**Abbreviations:** ATG, autophagy related; BafA1, bafilomycin A_1_; GFP, green fluorescent protein; HIP1R, huntingtin interacting protein 1 related; MEF, mouse embryo fibroblast; PIK3C2A, phosphatidylinositol-4-phosphate 3-kinase catalytic subunit type 2 alpha; SILAC, stable isotope labelling with amino acids in culture; SQSTM1, sequestosome 1; STRING, search tool for the retrieval of interacting genes/proteins

## Introduction

The catabolic macroautophagy pathway (“autophagy”, henceforth) describes the formation de novo of double-membrane-bound vesicles (autophagosomes) that sequester cargo for delivery to the lysosome for degradation. Autophagy occurs at low basal rates in healthy cells but is rapidly upregulated during nutrient/environmental stress. Coordinating the autophagosome assembly process is a family of autophagy related (ATG) proteins that work in concert to effect the lipidation of ubiquitin-like Atg8-family proteins (LC3 and GABARAP family members) at the nascent autophagic phagophore membrane (e.g. [1]). Atg8-family protein lipidation is controlled by two parallel conjugation systems. In the first, the ubiquitin-like protein ATG12 is constitutively conjugated to the structural protein ATG5, a step that requires ATG7 as the E1-like activating enzyme and ATG10 as the E2-like conjugating enzyme. ATG5 then binds to ATG16L1, forming the ATG12–ATG5-ATG16L1 complex, which acts as an E3-like lipid conjugation effector complex that recruits and stimulates ATG3, the E2-like conjugating enzyme in the second system. ATG3 regulates the transfer of PE to Atg8-family proteins, with ATG7 again acting upstream as the E1-like activating enzyme in this process [2]. Importantly, it is anticipated that lipids from diverse sources within the cell are incorporated into the expanding phagophore membrane, to meet the acute demand for membrane supply/turnover during nutrient stress [3, 4]. Candidate donor sources include: the ER [5]; ER exit sites/ERGIC [6]; the plasma membrane [7]; mitochondria [8]; COPII vesicles [9]; and the RAB11-positive recycling endosome [10]. Identifying the regulatory pathways that coordinate membrane delivery from these diverse sites remains a major challenge for the field.

Analysis of protein interaction networks within the autophagy pathway can indicate novel relationships between autophagosome intermediates and other organelles and/or membrane systems within the cell. Using multiple autophagy proteins as baits, Behrends et al. cataloged proteins comprising the autophagy interactome, revealing multiple novel network nodes linking autophagy with other key cellular pathways [11]. In particular, vesicular trafficking factors, including several RABs and RABGAPs, components of the TRAPPIII (trafficking protein particle III) complex, and key constituents of the clathrin complex were identified as candidate interactors of core autophagy molecules [11]. Subsequently, studies from other groups have highlighted roles for RABGAPs in autophagy [12], with examples including: TBC1D2A [13]; TBC1D14 [14, 15]; TBC1D25/OATL1 [16]; and TBC1D5 [17]. Similarly, components of the clathrin machinery have been shown to influence autophagy via the establishment of specific endosomal membrane pools incorporating the sole membrane-spanning core autophagy protein, ATG9, and/or ATG16L1 (e.g. [7, 17, 18]). Crucially, CLTC (clathrin heavy chain) has been reported to interact directly with ATG16L1 to coordinate its internalization into the endocytic compartment [7], while clathrin adaptors AP2A1 and AP2M1 have been shown to interact with ATG9 [17]. These findings complement the reported associations between clathrin complex components and Atg8-family proteins in proteomics datasets [11]. Linking these observations, TBC1D5 has been shown to act as a key determinant of ATG9 trafficking during autophagy, regulating the sorting of ATG9 away from the late endosome via differential associations with the retromer component VPS29 and the AP2 clathrin adapter complex [17]. TBC1D14 overexpression impairs autophagy through the generation of enlarged, tubulated recycling endosomes that retain the autophagy initiation kinase, ULK1 [14], and its influence on autophagy appears to be via binding to TRAPPIII—identified as an interactor of TECPR1 (tectonin beta-propeller repeat containing 1) by Behrends et al. [11]. Together, these proteins regulate RAB1-mediated release of ATG9 from the RAB11-positive compartment via the Golgi in a ULK1-independent manner to establish the ATG9-positive vesicular pool [15]. Efficient trafficking of ATG9A from the recycling endosome into pre-autophagic structures that are also positive for ATG16L1 and WIPI2 depends on sorting nexins SNX4 and SNX18, and DNM2 (dynamin 2) [19–21], while a role for ARFIP2/arfaptin-2 (ADP-ribosylation factor interacting protein 2) in mobilization of ATG9A-positive vesicles that deliver PI4KB/PI4KIIIbeta to the site of autophagosome assembly has also been described [22]. Notably RAB11A and transferrin receptor-positive compartments appear to be pivotal for autophagosome assembly, as here RAB11A binds WIPI2 to establish a platform for autophagosome assembly [10]. Clearly, the autophagy and intracellular membrane trafficking pathways are intimately linked, with routing of ATG9 through the endocytic system a prominent regulatory nexus [23, 24].

While there are numerous examples of how altered intracellular membrane trafficking pathways can impact on the autophagy response, the idea that certain autophagy proteins directly influence the specificity and/or efficiency of protein trafficking through the biosynthetic and/or endocytic routes has gained traction [25]. Autophagy proteins are implicated in unconventional protein secretion [26, 27] with cargoes including the inflammatory mediator IL1B/IL-1β [28] and the cystic fibrosis AAAF508 CFTR protein [29]. The autophagy machinery also influences the steady state distribution of a subset of surface proteins including the FAS receptor [30], the glucose transporter, SLC2A1/GLUT1 [31], and EGFR (epidermal growth factor receptor) [32]. Here, the involvement of autophagy in maintaining endosomal phosphatidylinositol-3-phosphate (PtdIns3P) levels and functional endocytic transport has been demonstrated [32]. Notably, surface levels of SLC2A1 were elevated in an autophagy-dependent manner in response to increased glucose demand through autophagy-mediated sequestration of TBC1D5 and subsequent diversion of SLC2A1 to the plasma membrane [31]. The autophagy machinery—exemplified by ATG5—also influences the properties of major histocompatibility complex (MHC) class II loading compartments [33, 34], and elevated surface levels of MHC class I have been reported in ATG5- and ATG7-deficient dendritic cells, caused by an internalization defect linked to differential mobility of AAK1 (AP2 associated kinase 1) [35]. From such studies, the concept of secretory autophagy has emerged [25], and indeed one report using *atg5^fl^°^x/fl^°^x^* mouse macrophages has provided evidence that a number of secreted, leaderless proteins (including FTH1 [ferritin heavy polypeptide 1] and several lysosomal proteins) have altered, relative extracellular abundances upon ATG5 ablation [36]. Based on these studies, a direct role for autophagosomes in the sequestration and delivery of secreted cargoes has been proposed [36]. With respect to endocytosis, and in addition to the aforementioned links between core autophagy proteins and the clathrin machinery, a novel pathway for association of Atg8-family proteins with endosomes has been described in the context of Aβ uptake in a mouse Alzheimer disease model [37]. This pathway of LC3-associated endocytosis (LANDO) requires ATG5 but not RB1CC1/FIP200 (necessary for canonical autophagy), and is also dependent on RUBCN/rubicon—a protein that restricts canonical autophagy [37].

Here, we have carried out unbiased quantitative proteomics analyses to characterise the ATG5 interactome in autophagy-competent and autophagy-incompetent cells, providing clear evidence for interactions between the lipidation machinery and components of clathrin-dependent membrane trafficking pathways. Indeed, analysis of clathrin adaptors present in our networks suggest that the AP2 and AP1 clathrin systems converge on ATG5. Within the clathrin endocytic machinery identified in the ATG5 interactome, we provide evidence that two putative ATG5 interactors—PIK3C2A (phosphatidylinositol-4-phosphate 3-kinase catalytic subunit type 2 alpha) and HIP1R (huntingtin interacting protein 1 related)—influence autophagic flux in cells in vitro. Finally, using surface biotinylation, we capture the surface proteomes of autophagy-competent and -incompetent cells under full media and starvation conditions, as a step towards understanding how the autophagy machinery influences conventional trafficking through the endocytic and secretory systems.

## Results and Discussion

### Quantitative ATG5 proteomics describes the hierarchical recruitment of autophagy regulators at the phagophore membrane.

**Figure 1.**
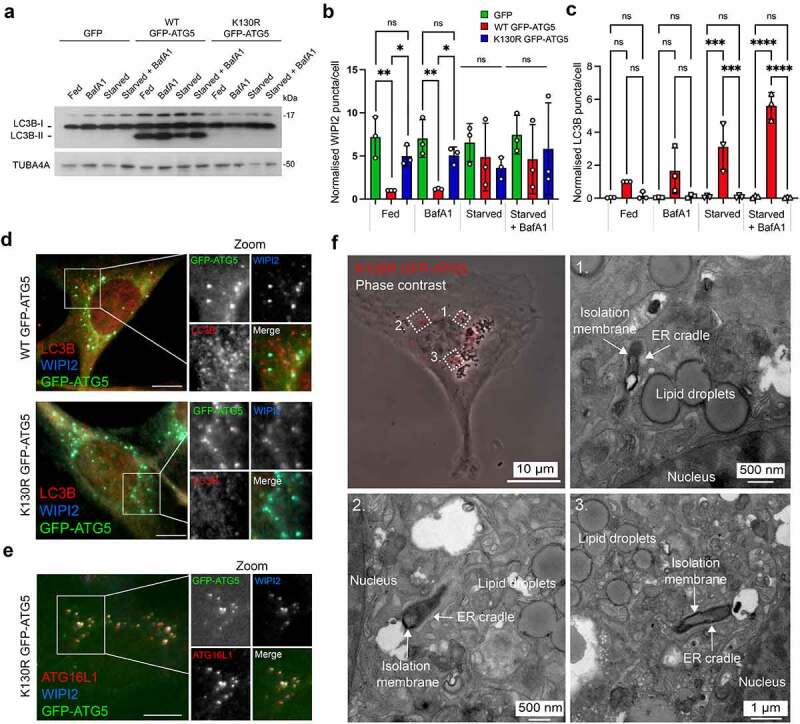
Characterization of the ATG5 cell-lines used for SILAC proteomics analysis of the ATG5 interactome. (**A**) Immunoblot of *atg5^−/−^* MEFs stably rescued with GFP, wild-type (WT) GFP-ATG5 or GFP-ATG5^K13^°^R^. Cells were incubated for 1 h in full-nutrient media or in HBSS (starvation), in the absence or presence of BafA1. Lysates were probed for LC3B and TUBA4A. Only the WT GFP-ATG5 rescued cells are capable of lipidating LC3B. (**B**) WIPI2 and (**C**) LC3B puncta analysis in fed and starved rescued MEFs, in the absence or presence of BafA1. Charts show mean ± SD; n = 3; 10 fields of cells per condition per experiment, normalized to WT GFP-ATG5 cells in the fed state; statistical significance calculated using ANOVA, followed by Tukey’s multiple comparison test. (***P < 0.001, **P < 0.01, *P < 0.05). (**D, E**) Markers of the early autophagosome colocalize with GFP-ATG5 in rescued MEFs. Example images of starved *atg5^−/−^* MEFs stably expressing WT GFP-ATG5 or GFP-ATG5^K13^°^R^, fixed then stained with antibodies against WIPI2 and LC3B (**D**), or WIPI2 and ATG16L1 (**E**). Bar: 10 µm. (**F**) CLEM analysis of starved *atg5^−/−^* MEFs stably rescued with GFP-ATG5^K13^°^R^. Panel top left shows a phase contrast image of a cell, with GFP-ATG5^K13^°^R^ puncta superimposed and false colored red. Panels 1-3 show example fields of areas depicted in the phase contrast image, with stalled phagophores.

To identify novel proteins that are recruited to the phagophore to facilitate membrane expansion and shaping, we used green fluorescent protein (GFP) affinity immunoisolation (GFP-Trap) coupled with SILAC (stable isotope labelling with amino acids in culture)-based quantitative proteomics to define the ATG5 interactome. We used lentiviruses to stably “rescue” *atg5^−/−^* mouse embryo fibroblasts (MEFs) with: (i) GFP; (ii) wild-type GFP-ATG5 (WT GFP-ATG5); or (iii) GFP-ATG5^K13^°^R^ (a point mutation that prevents conjugation to ATG12 [38]). These constructs were chosen to enable comparative analyses of the interactomes of conjugation competent and conjugation deficient ATG5. In *atg5^−/−^* MEFs, MAP1LC3B/LC3B lipidation was absent as expected, and the levels of SQSTM1/p62 (a protein that is degraded by autophagy), were constitutively high (**Fig. S1A**). Consistent with this, LC3B puncta were dramatically reduced, even when cells were starved in the presence of bafilomycin A_1_ (BafA1), while WIPI2 puncta numbers were elevated even under basal conditions (**Fig. S1B-D**). Rescued MEFs were validated for their responses to amino acid/growth factor starvation (1 h), and as anticipated, only the WT GFP-ATG5 cell-line supported LC3B lipidation (Fig. 1A), showed normal WIPI2 puncta responses (Fig. 1B), and assembled LC3B-decorated autophagosomes (Fig. 1C). In both the WT GFP-ATG5 and GFP-ATG5^K13^°^R^ cell-lines, WIPI2 colocalized with GFP-ATG5 at presumed autophagosome assembly sites during starvation (Fig. 1D). ATG16L1 also localized at these sites in the autophagy-incompetent GFP-ATG5^K13^°^R^ cells, suggesting that its targeting is not dependent on ATG12 conjugation to ATG5 (Fig. 1E). In GFP-ATG5^K13^°^R^ cells, GFP-labelled structures were confirmed as stalled phagophores—flattened, electron-dense membrane structures cradled by characteristic ER tubules [39, 40]—using correlative light and electron microscopy (CLEM) (Fig. 1F).

**Figure 2.**
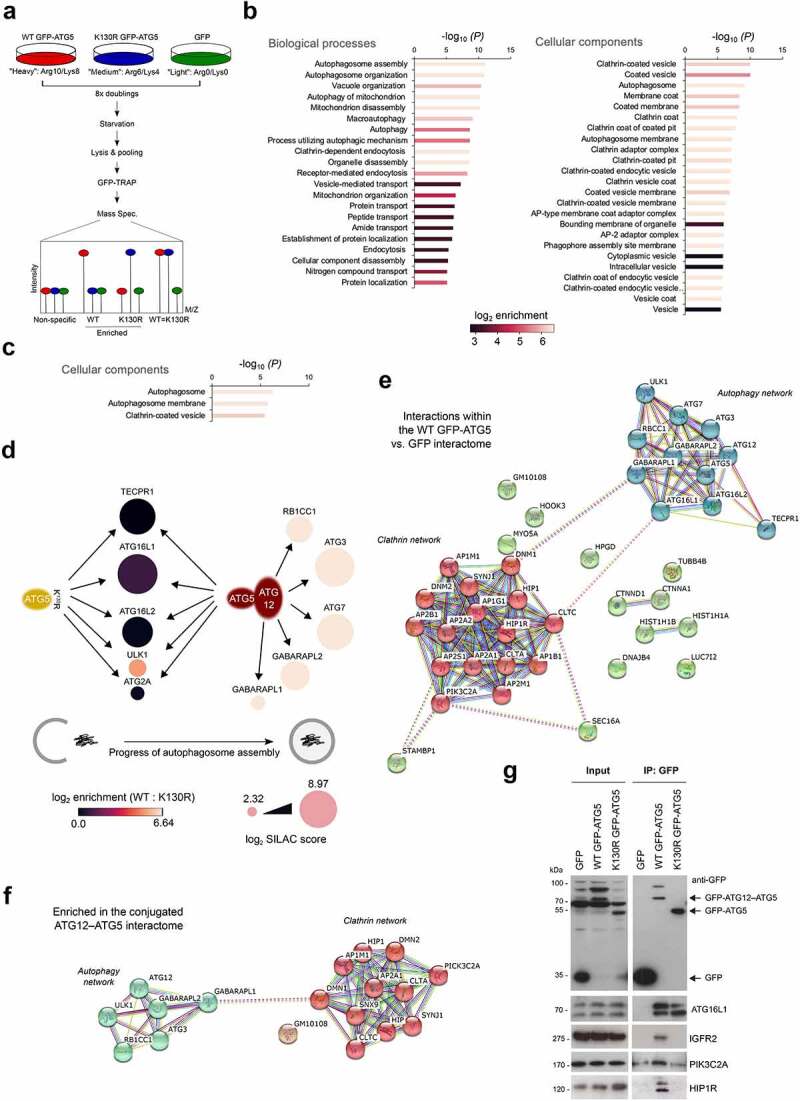
Analysis of the ATG5 interactome. (**A**) Schematic of the SILAC protocol for enrichment of ATG5 interacting proteins. (**B**) Gene ontology analysis of the WT GFP-ATG5 interactome (>2-fold enrichment over GFP). Interacting protein families are separated into biological processes (left) and cellular components (right), with bars colored for log_2_ enrichment. (**C**) Gene ontology analysis of the conjugated ATG12–ATG5 interactome (>2-fold enrichment over GFP-ATG5^K13^°^R^). (**D**) Schematic of the autophagy protein interactions identified in the WT GFP-ATG5 and GFP-ATG5^K13^°^R^ interactomes (vs. GFP). Circles representing proteins are sized by log_2_ SILAC “score” as an indicator of confidence within the dataset, and are shaded according to log_2_ enrichment of WT GFP-ATG5 vs. GFP-ATG5^K13^°^R^. (**E**) STRING analysis of the high confidence WT GFP-ATG5 vs. GFP interactome showing 2 distinct interaction hubs: autophagosome biogenesis and clathrin-mediated vesicular trafficking. (**F**) STRING analysis of the high confidence conjugated ATG12–ATG5 interactome. (**G**) Immunoblots of selected candidate interactors. GFP-TRAP immunoisolates were prepared from GFP, WT GFP-ATG5 and GFP-ATG5^K13^°^R^ MEFs following1 h starvation.

Each MEF cell-line was then grown in SILAC media for 8 cell doubling events, before being starved for 1 h, and then lysed for GFP-Trap immunoisolation (Fig. 2A). Samples were analyzed by mass spectroscopy, and a Sequest search carried out against the Uniprot mouse database with a <5% FDR cut-off applied to eliminate low confidence hits (see **Materials and Methods**). Positive interactors were identified as those that were present in the WT GFP-ATG5 interactome but were absent in the GFP equivalent (2 or more individual peptides; >2-fold enrichment over GFP), and the obtained dataset comprised of 495 proteins (**Table S1** showing representative data from 2 identical experiments). Of these, 218 proteins were also enriched >2-fold over the GFP-ATG5^K13^°^R^ dataset, and these represented candidate ATG12–ATG5 conjugate interactors (**Table S2**). In the GFP-ATG5^K13^°^R^ dataset there were 364 proteins enriched >2-fold over GFP, of which 127 proteins also showed a >2-fold enrichment over WT GFP-ATG5, and these represented candidate unconjugated ATG5 interactors (**Table S3**). Ontology analysis confirmed enrichment of proteins involved in autophagosome assembly, alongside other proteins acting in additional membrane trafficking pathways, particularly clathrin-mediated endocytosis (see below; Fig. 2B**, C**).

Analysis of autophagy proteins identified in the WT GFP-ATG5 and GFP-ATG5^K13^°^R^ datasets pointed to a hierarchical recruitment of autophagosome assembly factors in autophagy competent and incompetent cells, which broadly agreed with expected recruitment dynamics at autophagosome assembly sites [41, 42] (Fig. 2D). For example, proteins acting during early stages of autophagosome assembly were detected >2-fold over GFP in the unconjugated ATG5 dataset (see **Table S2**; e.g., ATG16L1; ATG2A; ULK1); whereas proteins that were enriched in the ATG12–ATG5 conjugate interactome (**Table S2**) also included those linked to the latter stages of autophagosome maturation and Atg8 lipidation (e.g., ATG3, ATG7, GABARAPL1, GABARAPL2/GATE-16) (Fig. 2D). Several other Atg8 family members were detected in the SILAC datasets, but below the peptide number threshold (namely: GABARAP; LC3A; LC3B; data not shown).

Unexpectedly, TECPR1 was found to be a strong interactor in both WT GFP-ATG5 and GFP-ATG5^K13^°^R^ datasets (Fig. 2D; **Table S2**). TECPR1 binds directly to the ATG12–ATG5 conjugate to facilitate autophagosome-to-lysosome fusion [43], but our interactions data suggest possible further roles during early stages of autophagosome formation. Consistent with this, TECPR1 has been observed to colocalize with ATG5 at presumed autophagosome assembly sites in co-overexpressing cells [43]. It was also notable that ATG16L1 was enriched in both the WT GFP-ATG5 and GFP-ATG5^K13^°^R^ datasets (Fig. 2D; **Table S2**), as this was consistent with the observed co-localization of ATG16L1 at assembly sites in GFP-ATG5^K13^°^R^ MEFs (Fig. 1E). Indeed, ATG16 has been found to bind directly to ATG5 [44] to reinforce its lipid binding capabilities [45], although yeast Atg16 is dispensable for the E3-like activity of the ATG12–ATG5 conjugate in vitro [2]. We also detected ATP6V0D1 (ATPase, H+ transporting, lysosomal V0 subunit D1)—a component of the V_0_ domain of the V-ATPase in both WT and GFP-ATG5^K13^°^R^ interactomes (**Table S2**), which is consistent with recent descriptions of ATG16L1 and ATG5 interacting with the ATP6V1A (ATPase, H+ transporting, lysosomal V1 subunit A) component during non-canonical LC3 lipidation pathways [46] and during bacterial infection [47]. Of further note, GFP-ATG5^K13^°^R^ also interacted strongly with ATG16L2—an isoform of mammalian ATG16 that binds to ATG12–ATG5 but cannot support autophagosome assembly due to variations in the coiled coil central section [48]. RBCC1 (RB1-inducible coiled-coil 1) was only highlighted in the ATG12–ATG5 conjugate dataset [Fig. 2D; **Table S2**]), meanwhile other components of the mammalian ULK1 complex (namely, ATG13 and ATG101) were not found in any of the datasets. Additional autophagosome assembly factors that were detected below the 2-fold enrichment threshold over GFP in the WT GFP-ATG5 dataset included: ATG2B; ATG10; SQSTM1/p62; FYCO1 (FYVE and coiled-coil domain containing 1); WDR45B/WIPI3; WDR45/WIPI4 (data not shown).

### Enrichment of the clathrin trafficking machinery in the GFP-ATG5 interactomes.

In the WT GFP-ATG5 interactome, there was a substantial enrichment of proteins with roles in endocytic pathways, in particular, clathrin-mediated vesicular trafficking (Fig. 2B**, C; Table S2**). These proteins were largely absent in the unconjugated ATG5 dataset (**Table S3**), suggesting that ATG12–ATG5 conjugation is needed for this interaction (examples included: CLTC; CLTA [clathrin, light polypeptide (Lca)]; SNX9 [sorting nexin 9]; HIP1 [huntingtin interacting protein 1]; HIP1R; GAK [cyclin G associated kinase]; DNMBP [dynamin binding protein]; AAK1 [AP2 associated kinase 1]; EPS15 [epidermal growth factor receptor pathway substrate 15]; PIK3C2A). High confidence interactome lists were next assembled to include only those proteins that were >8-fold enriched, with >7.5 Score values. These were subjected to STRING analysis to visualize relationships within protein families (Fig. 2E, F), from which, two clearly separate interactions groups emerged: (i) core autophagosome assembly proteins; (ii) regulators of clathrin-dependent vesicular trafficking (Fig. 2E). In addition to clathrin heavy and light chains, a number of clathrin adaptor subunits of the AP1 (B1, M1, G1) and AP2 (A1, A2, B1, M1) classes were identified (Fig. 2E). For comparison, we also assembled a comparative ATG12–ATG5 conjugate dataset based on >8-fold enrichment vs. GFP-ATG5^K13^°^R^, with >7.5 Score value (Fig. 2F). Once again, 2 distinct, albeit more restricted clusters of autophagy and clathrin networks were evident, arguing that interactions within both groups are favoured following ATG5 conjugation to ATG12. By contrast, aside from the relatively small number of autophagy proteins that were present, the only other possible protein grouping that emerged from parallel GFP-ATG5^K13^°^R^ dataset STRING analysis included components of the extracellular matrix (**Fig. S2**).

The identification of endocytic proteins in the control of membrane phosphoinositide identity (PIK3C2A; SYNJ1 [synaptojanin 1]), clathrin recruitment/activity during coat assembly/scaffolding (PIK3C2A; SNX9; PICALM [phosphatidylinositol binding clathrin assembly protein]; AAK1; EPS15), uncoating (GAK), actin regulation (HIP1R), and vesicle deformation/scission (DNM1 [dynamin 1]; DNM2 [dynamin 2]; SNX9), suggested that ATG5 interacts with the clathrin-associated endocytic machinery [49]. The presence of AP1 adaptor components also pointed to a possible relationship between ATG5 and the Golgi-to-early/recycling endosome trafficking step [50]; meanwhile the enrichment of IGF2R/CI-M6PR (insulin-like growth factor 2 receptor), argued for a more global role in clathrin-mediated trafficking within the cell. Detection of CLTC in ATG5 interaction data here and in Behrends et al. [11] was of particular interest since previous research has shown that ATG16L1 can be co-isolated with CLTC (and AP2), and that CLTC contributes to autophagosome biogenesis via the establishment of a distinct ATG16L1-positive endocytic membrane compartment [7, 18]. How the ATG12–ATG5 conjugate contributes to this process remains uncertain. Experimental data suggest that the N-terminal domain of ATG16L1 is responsible for the CLTC interaction, but given that the ATG12–ATG5 conjugate also binds to ATG16L1 in this region, it is uncertain how ATG12–ATG5 influences this association (although overexpression of ATG5 has been found to not disturb the ATG16L1-CLTC interaction) [7]. Its presence in both our WT GFP-ATG5 and GFP-ATG5^K13^°^R^ datasets, suggests that binding to ATG16L1 is not contingent on ATG12 conjugation (**Table S2**). Since CLTC, and indeed the large majority of clathrin-associated proteins were absent in the GFP-ATG5^K13^°^R^ interactome (**Table S3**), ATG5 engagement with the clathrin machinery is unlikely to occur via ATG16L1.

### Validation of candidate ATG12–ATG5 interactors

We next sought to validate selected candidate interactors enriched in the WT GFP-ATG5 interactome, namely: PIK3C2A, HIP1R, IGFR2, SNX9, MYO5A (myosin VA), DNM1, DNM2 (Fig. 2G). GFP-Trap affinity isolates of lysates from the GFP, WT GFP-ATG5, and GFP-ATG5^K13^°^R^-expressing *atg5^−/−^* MEFs were analyzed by immunoblotting. This clearly showed the presence of ATG16L1 in both WT GFP-ATG5 and GFP-ATG5^K13^°^R^ affinity isolates (Fig. 2G), as predicted from the SILAC-based proteomics analysis (**Table S2**). By contrast, PIK3C2A, HIP1R and IGFR2 were strongly enriched in the WT GFP-ATG5 affinity isolation lysate (Fig. 2G). However, MYO5A, DNM1, DNM2, and SNX9 could not be detected in immunoblots of affinity isolates from either WT or GFP-ATG5^K13^°^R^ cells (**data not shown**).

To establish whether the link between ATG12–ATG5 and the clathrin system is dependent upon productive autophagosome assembly, we established cell-lines stably expressing GFP, WT GFP-ATG5 or GFP-ATG5^K13^°^R^ in an *atg3^−/−^* MEF background [51] for SILAC-based proteomics (**Fig. S3**). As expected, *atg3^−/−^* MEFs were deficient in LC3B lipidation and autophagy, even when supplemented with the ATG5 constructs (**data not shown**). Quantitative proteomics was carried out following nutrient starvation as before; proteins with a peptide count <2 were removed, and a WT GFP-ATG5 interactome was constructed comprising proteins that were >2-fold enriched over GFP and GFP-ATG5^K13^°^R^ (**Tables S4, S5**). Overall, the comparable level of enrichment for WT GFP-ATG5 interactors identified in the *atg3^−/−^* background was lower than in the *atg5^−/−^* background, perhaps because GFP-ATG5 was expressed alongside the endogenous protein. Core autophagy proteins that were enriched in the WT GFP-ATG5 interactome in *atg3* null MEFs included: GABARAPL2/GATE-16, ATG7, ATG12, TECPR1, ATG16L1, and ATG16L2 (ATG3 peptides detected are likely to be the truncated product of the mutant ATG3 allele generated by targeting exon 10 [51]). Also identified were several proteins implicated in the regulation of clathrin-mediated trafficking, including: CLTC, CLTA, HIP1R, SNX9, DNM1, DNM2, and PIK3C2A (**Table S4**). STRING analysis of this dataset once again revealed 2 clusters centred on autophagy and clathrin-mediated endocytic trafficking (**Fig. S3A**). Similar analysis of the GFP-ATG5^K13^°^R^ interactome once again only showed evidence of a limited cluster of autophagy proteins (**Fig. S3B**). In this background, immunoblotting confirmed that PIK3C2A co-isolated only in WT GFP-ATG5 pull-downs (**Fig. S3C**). This argues that ATG12–ATG5 can still engage with clathrin-associated trafficking factors regardless of the requirement for progressive autophagosome assembly and the accompanying demand for membrane supply at autophagosome assembly sites.

### PIK3C2A and HIP1R contribute to basal and starvation-induced autophagy

To determine the possible relevance of interactions between the ATG12–ATG5 conjugate and proteins linked to clathrin-mediated endocytosis, we analyzed the autophagy responses in cells lacking either PIK3C2A or HIP1R. Phosphatidylinositol 3-kinases (PtdIns3Ks) and phosphoinositide 3-kinases are lipid kinases involved in a large set of biological processes, including endocytic trafficking, receptor signalling, organization of the cytoskeleton, and autophagy [52, 53]. There are three such classes and 8 such proteins in total, all of which phosphorylate the D3 position of the inositol ring of phosphatidylinositol (PtdIns) to generate PtdIns3P or other phosphoinositides [54], with the sole class III member, PIK3C3/VPS34, being responsible for a major fraction of PtdIns3P within cells needed during autophagosome formation [55]. Of the less well characterized class II phosphoinositide 3-kinases (PIK3C2A, PIK3C2B, PIK3C2C), PIK3C2A can also form phosphatidylinositol-3,4-bisphosphate (PtdIns[3,4]P_2_ [56], a lipid that is concentrated at the plasma membrane and is required for the nucleation of clathrin-coated pits [e.g. [57]]). At the plasma membrane, PIK3C2A spatiotemporally controls clathrin-mediated endocytosis by regulating clathrin-coated pit maturation [56]. PIK3C2A plays a crucial role in development, as loss of the *Pik3c2a* gene in mouse causes early embryonic lethality due to defective vascular development [58], and homozygous loss-of-function mutations have recently been identified in patients with broad-ranging developmental abnormalities linked to ciliary defects [59]. Indeed, PIK3C2A influences cilia formation by regulating sonic hedgehog signalling [60]. In addition to these roles, data suggest that PIK3C2A may have a role in autophagy: it was identified as an interactor of ATG7 (but not ATG5) in the Behrends et al. study [11], and a reduction in LC3 puncta numbers has been observed when PIK3C2A is silenced together with PIK3C2B [52]. This prompted us to further explore the involvement of PIK3C2A in autophagy control.

**Figure 3.**
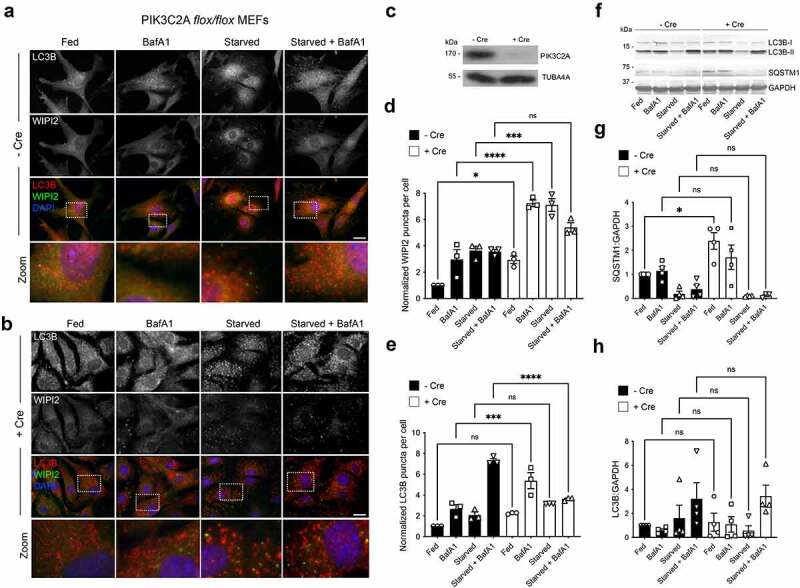
Autophagic flux deficiencies in *pik3c2a* null cells. (**A, B**) Immunofluorescence images of *pik3c2a^fl^°^x/fl^°^x^* MEFs under full nutrients or starved, in the absence or presence of BafA1, without (**A**) and with (**B**) addition of Cre recombinase. Cells were fixed then stained with antibodies against LC3B (red) and WIPI2 (green). DAPI staining is in blue. Bar: 10 µm. (**C**) Immunoblot of *pik3c2a^fl^°^x/fl^°^x^* MEFs showing loss of PIK3C2A following incubation with Cre recombinase. WIPI2 (**D**) and LC3B (**E**) puncta quantification in fed or starved cells in the absence or presence of BafA1. Mean ± SD; n = 3; 10 fields of cells per condition per experiment, normalized to control/fed conditions; statistical significance calculated using ANOVA, followed by Tukey’s multiple comparison test. (***P < 0.001, **P < 0.01, *P < 0.05). (**F-H**) Immunoblot analysis of SQSTM1 levels and LC3B lipidation status in *pik3c2a^fl^°^x/fl^°^x^* MEFs under full nutrients or starved, in the absence or presence of BafA1. Example immunoblot (**F**), and densitometry quantification for LC3B-II:LC3B-I (**G**) and SQSTM1 (**H**). Mean ± SD; n = 3-4 immunoblots, normalized to GAPDH (SQSTM1 blots) and fed condition -Cre; statistical significance calculated using one-way ANOVA, followed by Tukey’s Range test. (*P < 0.05).

*pik3c2a^fl^°^x/fl^°^x^* MEFs [58] were used to assess the formation of autophagosomes in basal and starvation conditions (Fig. 3). Immunofluorescence staining for WIPI2 and LC3B was carried out with or without addition of Cre recombinase (48 h), which caused efficient ablation of PIK3C2A expression (Fig. 3A-C). Quantification revealed that removal of PIK3C2A led to a significant increase in WIPI2 puncta numbers in full-nutrient conditions, with a further marked increase when cells were treated with BafA1 or starved in the absence or presence of BafA1 (Fig. 3D). In Cre-treated cells, LC3B puncta numbers were significantly increased only in fed cells in the presence of BafA1, with an absence of the expected starvation-induced LC3B puncta increases in the absence and presence of BafA1 (Fig. 3E). These data are consistent with an impairment at the autophagosome assembly and/or LC3B lipidation stage (Fig. 3D, E). Immunoblotting for LC3 lipidation and SQSTM1 turnover (Fig. 3F-H) did confirm differences in the relative levels of LC3-II between fed or starved cells treated or not with Cre (Fig. 3G), probably reflecting sensitivity differences between the 2 assays, although basal SQSTM1 levels were significantly higher in cells lacking PIK3C2A (Fig. 3H). Together, these data suggest autophagic flux deficiencies with reduced substrate (e.g., SQSTM1) turnover in the fed state.

**Figure 4.**
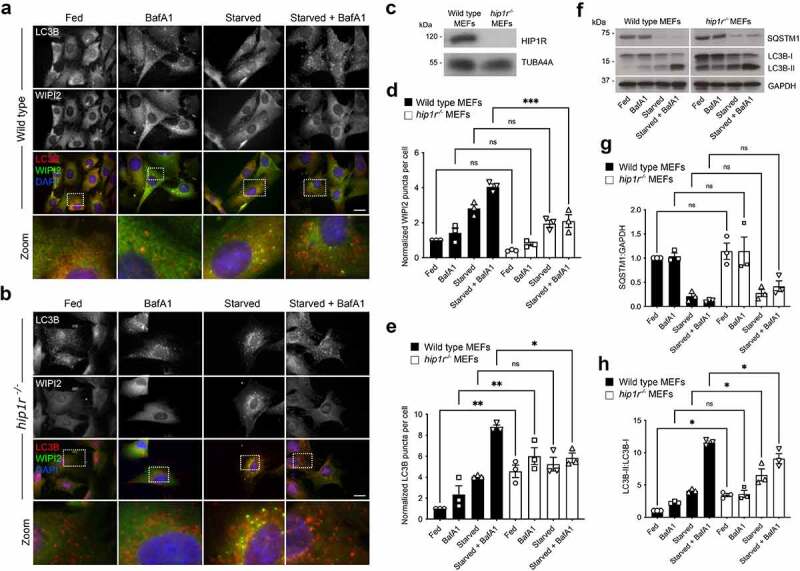
Autophagic flux deficiencies in *hip1r* null cells. (**A-B**) Immunofluorescence images of wild-type (**A**) and *hip1r^−/−^* (**B**) MEFs under full nutrients or starved, in the absence or presence of BafA1. Cells were fixed then stained with antibodies against LC3B (red) and WIPI2 (green). DAPI staining is in blue. Bar: 10 µm. (**C**) Immunoblot of wild-type and *hip1r^−/−^* MEFs. WIPI2 (**D**) and LC3B (**E**) puncta quantification in fed or starved cells in the absence or presence of BafA1. Mean ± SEM; n = 3; 10 fields of cells per condition per experiment; statistical significance calculated using ANOVA, followed by Tukey’s Range test. (***P < 0.001, **P < 0.01, *P < 0.05). (**F-H**) Immunoblot analysis of SQSTM1 levels and LC3B lipidation status in wild-type and *hip1r* null MEFs grown in full nutrients or starved, in the absence or presence of BafA1. Example immunoblot (**F**), and densitometry quantification for SQSTM1 (**G**) and LC3B-II:LC3B-I (**H**). Mean ± SD; n = 3 immunoblots, normalized to GAPDH (SQSTM1 blots) and wild-type cells in fed condition; statistical significance calculated using one-way ANOVA, followed by Tukey’s multiple comparison test. (*P < 0.05).

HIP1R is one of a number of essential adaptor proteins that regulate clathrin-mediated endocytosis. It is a component of clathrin-coated pits, recruited by CLTA, via its central dimerization-competent coiled-coil region, and binds/regulates local actin filament assembly [61, 62]^,^ [63-66]. HIP1R has an N-terminal ANTH (AP180 N-terminal homology) domain, through which it interacts with phospholipids [67, 68], and it has also been shown to have a role in vesicular trafficking at the *trans*-Golgi network (TGN) [69]. Since both the clathrin-mediated endocytic system and the actin cytoskeleton contribute to autophagosome assembly [7, 70], HIP1R may couple these systems to the autophagy machinery via ATG12–ATG5. Wild-type (WT) and *hip1r* null MEFs (kindly provided by Dr. Theodora Ross; UT Southwestern Medical Centre) were used to assess the contribution of HIP1R during basal and starvation-induced autophagy by immunofluorescence staining for WIPI2 and LC3B (Fig. 4). The autophagy response of *hip1r* null cells was indicative of a block in autophagic flux: in basal conditions, LC3B puncta numbers were significantly higher than in WT MEFs, but did not increase substantially in the presence of BafA1 (Fig. 4A, E). No further increase was recorded following nutrient starvation in the absence of BafA1, however in the presence of BafA1 there were significantly fewer LC3B puncta numbers compared to wild-type cells (Fig. 4A, E). WIPI2 puncta numbers in *hip1r* null MEFs followed a similar pattern to WT cells, although in the starved + BafA1 condition WIPI2 puncta numbers were significantly lower in *hip1r* null cells (Fig. 4A, D). Immunoblotting suggested no differences in the basal levels and turnover of SQSTM1 in WT and *hip1r^−/−^* MEFs (Fig. 4F, G); however, levels of lipidated LC3-II were significantly higher in the *hip1r^−/−^* cells in basal and starvation conditions, and were significantly lower in cells starved in the presence of BafA1 (Fig. 4F, H). In summary, while SQSTM1 turnover as measured by immunoblotting did not reveal differences between cell-lines, lipidated LC3-II levels were broadly consistent with the imaging data in that in *hip1r^−/−^* cells demonstrated impaired autophagosomal flux.

### ATG5 loss of function and general endocytic trafficking

To test whether a functional ATG12–ATG5 conjugate influences general endocytic activity, we measured fluorescent (Alexa 647) transferrin uptake in the three MEF cell-lines by flow cytometry (Fig. 4A). Over a period of 40 min, internalized transferrin fluorescence intensity increased in each of the cell-lines as expected, but with no significant differences between them at any time-point (Fig. 4A). This indicated that bulk endocytosis was not affected by autophagy pathway activity and the availability of ATG12–ATG5 in this context. Analysis of the passage of endocytosed material into acidic compartments was then carried out using the pHrodo dye. Reconstituted *atg5^−/−^* MEFs (GFP, WT GFP-ATG5, GFP-ATG5^K13^°^R^) were incubated for 30 min at 37°C with pHrodo, and fluorescence was measured in live cells under fed and starvation conditions by confocal microscopy (Fig. 4B-E**)**. Initial fluorescence levels did not differ significantly between cell-types either in full media or under starvation conditions, suggesting equivalent dye uptake (Fig. 4B, D); however, we did observe time-dependent differences in the relative fluorescence signals of the WT and GFP-ATG5^K13^°^R^ reconstituted MEFs following wash on comparison to GFP expressing cells (Fig. 4C, E). In fed conditions, pHrodo fluorescence levels gradually increased in autophagy-active WT GFP-ATG5 MEFs, whereas in GFP-ATG5^K13^°^R^ MEFs, relative fluorescence declined over time suggesting inefficient entry into acid compartments and signal degradation (Fig. 4C). By contrast pHrodo fluorescence increased steadily in both WT and GFP-ATG5^K13^°^R^ reconstituted *atg5^−/−^* MEFs during starvation relative to GFP cells, with fluorescence levels increasing significantly faster in GFP-ATG5^K13^°^R^ MEFs (Fig. 4E). These data hint at involvement of ATG5 in conjugatable or non-conjugatable in influencing the kinetics of endocytic cargo flux, dependent on nutrient availability.

### Quantitative SILAC cell surface proteomics and the ATG12–ATG5 conjugate

The proteomics data linking ATG12–ATG5 and the clathrin-mediated vesicle trafficking machinery, and the unexplained differences in flux through the endocytic network between the reconstituted MEFs, prompted us to test whether the steady state surface proteome is influenced by the actions of the ATG12–ATG5 conjugate in supporting a functional autophagy system. We used biotinylation to capture the surface proteomes of the reconstituted *atg5^−/−^* MEFs (GFP, WT GFP-ATG5, GFP-ATG5^K13^°^R^) under fed and starved conditions for SILAC-based proteomics (respectively, 4 and 3 independent surface biotinylation experiments). Following data sampling to exclude low confidence peptides, and data normalization to account for variabilities in labelling efficiency (see **Materials and Methods**), proteins that were represented in at least 3 of the 4 repeats were identified and their relative abundancies compared between the three different cell-lines (**Tables S6-S9**).

**Figure 5.**
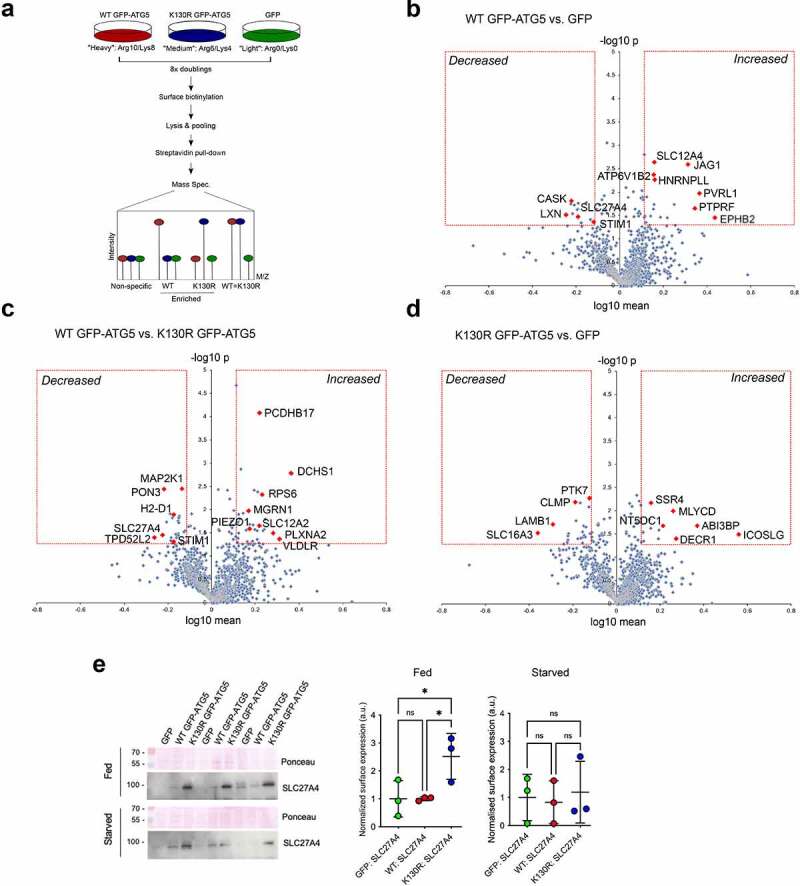
Surface protein abundances in *atg5^−/−^* MEF rescued with GFP, WT GFP-ATG5 or GFP-ATG5^K13^°^R^. (**A**) Schematic of the SILAC protocol for enrichment of biotinylated surface proteins in the rescued *atg5^−/−^* MEF cell lines. (**B-D**) Pairwise comparisons of surface protein abundances analyzed by surface biotinylation-streptavidin affinity isolation and SILAC quantitative proteomics in fed conditions, plotting log_10_ mean fold-change against -log_10_ *p* value (T-test), for the following combinations: (**B**) WT GFP-ATG5 vs. GFP; (**C**) WT GFP-ATG5 vs. GFP-ATG5^K13^°^R^; (**D**) GFP-ATG5^K13^°^R^ vs. GFP. The red boxes indicate those proteins whose levels were either increased or decreased above an arbitrary 1.3-fold cut-off, with statistical significance p<0.05. Proteins that fall into these categories are listed in **Tables S7-S9**. Red diamonds depict selected proteins of potential interest. (**E**) Immunoblot analysis of surface SLC27A4 in the rescued *atg5^−/−^* MEF cell-lines under fed and starved conditions. Densitometry measurements were normalized to Ponceau intensity, and are depicted relative to the GFP-expressing MEF mean value.

Data obtained from cells under full nutrient conditions were displayed as volcano plots (log10 mean vs. -log10 p-value; 1.3-fold cut off for enrichment; Fig. 5A-D). Individual proteins that were consistently altered >1.3-fold in the WT GFP-ATG5 vs. GFP dataset included: EPHB2 (Eph receptor B2), NECTIN1/PVRL1, PTPRF (protein tyrosine phosphatase, receptor type, F), JAG1 (jagged 1); HNRNPLL (heterogeneous nuclear ribonucleoprotein L-like), ATP6V1B2 (ATPase, H+ transporting, lysosomal V1 subunit B2), SLC12A4 (solute carrier family 12, member 4) (all up); and LXN (latexin), STIM1 (stromal interaction molecule 1); CASK; SLC27A4/FATP4 (solute carrier family 27 (fatty acid transporter), member 4) (all down) (Fig. 5B**; Tables S6, S7**). Examples of proteins that differed between the WT GFP-ATG5 and GFP-ATG5^K13^°^R^ datasets included: DCHS1 (dachsous cadherin related1), VLDLR (very low density lipoprotein receptor), PLXNA2 (plexin A2), SLC12A2 (all up); and STIM1, TPD52L2 (tumor protein D52-like 2), PON3 (paraoxanase 3), SLC27A4, H2-D1 (histocompatibility 2, D region locus 1), MAP2K1 (mitogen-activated protein kinase kinase 1) (all down) (Fig. 5C**; Tables S6, S8**). Comparing the GFP-ATG5^K13^°^R^ and GFP surface proteomes revealed differences in levels of: ICOSL/ICOSLG (icos ligand), ABI3BP (ABI family member 3 binding protein), DECR1 (2,4-dienoyl-CoA reductase 1, mitochondrial), MLYCD (malonyl-CoA decarboxylase), NT5DC1 (5’-nucleotidase domain containing 1), SSR4 (signal sequence receptor, delta) (all up); and the monocarboxylate transporter SLC16A3/MCT4, LAMB1 (lamin B1), CLMP (CXADR-like membrane protein), PTK7 (PTK7 protein tyrosine kinase 7) (all down) (Fig. 5D**; Tables S6, S9**). SLC27A4 is a member of the fatty acid transport protein family with roles in regulated fatty acid uptake [71], and its relative enrichment on the surface of autophagy-deficient cells might be expected in cells compensating for the lack of autophagy-mediated fatty acid mobilization; meanwhile, recent data suggest a possible link between SLC27A4 and ATG4B in lung cancer cells [72]. We assessed surface SLC27A4 levels by immunoblotting, and this revealed elevated surface SLC27A4 in GFP-ATG5^K13^°^R^ MEFs compared with both GFP and WT GFP-ATG5 expressing cells (Fig. 5E). This confirmed the SILAC data for elevated SLC27A4 levels in GFP-ATG5^K13^°^R^ cells vs. WT GFP-ATG5 cells (Fig. 5C), but the immunoblotting and SILAC data were not consistent when comparing WT GFP-ATG5 and GFP cells (Fig. 5B; SLC27A4 did not reach detection threshold for 3 from 4 SILAC repeats in the comparable GFP-ATG5^K13^°^R^ vs. GFP datasets, see **Table S6**).

**Figure 6.**
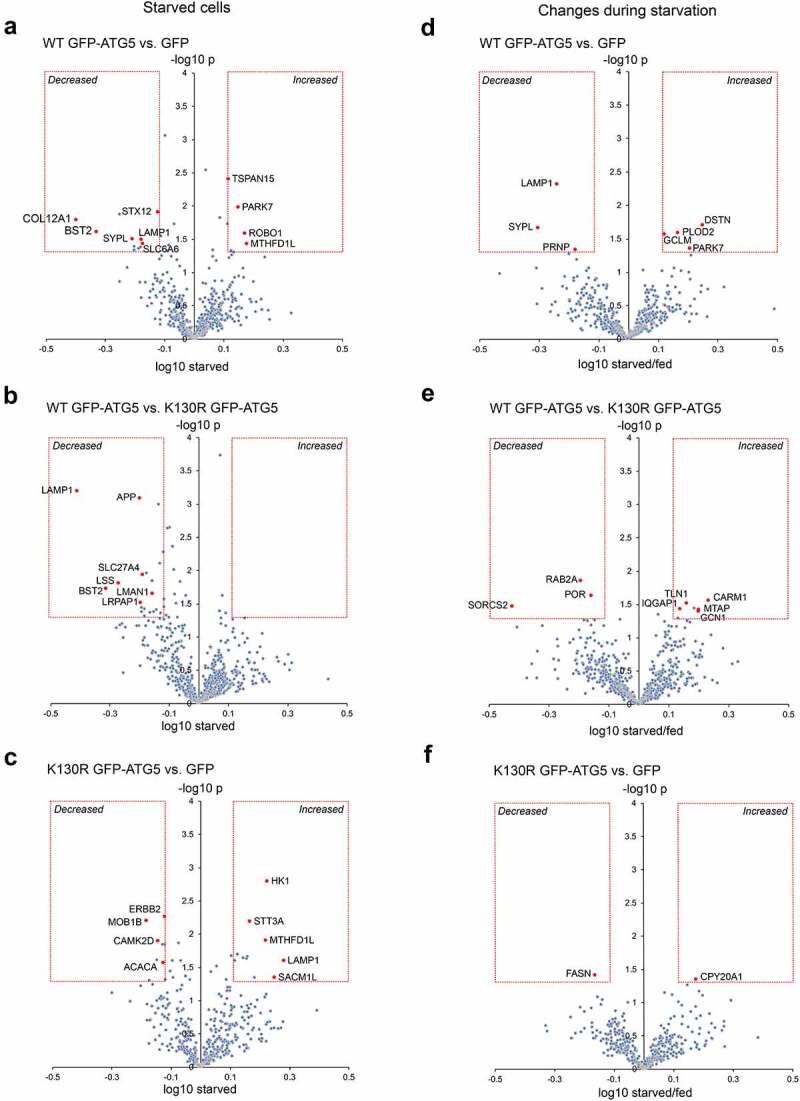
Surface protein abundances during starvation in *atg5^−/−^* MEF rescued with GFP, WT GFP-ATG5 or GFP-ATG5^K13^°^R^. Pairwise comparisons of surface protein abundance analyzed by surface biotinylation/streptavidin pull-down and SILAC quantitative proteomics in starvation conditions (1 h) (**A-C**), and changing surface protein abundances from fed to starvations conditions (**D-F**). Pairwise surface protein expression analyses: (**A, D**) WT GFP-ATG5 vs. GFP; (**B, E**) WT GFP-ATG5 vs. GFP-ATG5^K13^°^R^; (**C, F**) GFP-ATG5^K13^°^R^ vs. GFP. (**D-F**) To indicate changes in surface expression between fed and starved states, x-axes depict log_10_ ratio of starved/fed values. In all examples, the red boxes indicate 1.3-fold enrichment cut-off, with statistical significance p<0.05 (T-test). Proteins that fall into these categories are listed in **Tables S10, S11**. Red diamonds depict selected proteins of potential interest.

We next analyzed the surface proteomes of cells subjected to 1h nutrient starvation (Fig. 6A**-C; Tables S10, S11**). From the 3 proteomics datasets obtained, 494 (WT GFP-ATG5 vs. GFP), 609 (WT GFP-ATG5 vs. GFP-ATG5^K13^°^R^) and 409 (GFP-ATG5^K13^°^R^ vs. GFP) proteins were represented on each occasion (**Table S10**). Of those, proteins whose levels were significantly changed >1.3-fold on the surface of WT GFP-ATG5 over GFP-expressing cells included: ROBO1, PARK7, TSPAN15, MTHFD1L (all up); and COL12A1 (collagen, type XII, alpha 1), BST2 (bone marrow stromal cell antigen 2), SYPL (synaptophysin-like protein), LAMP1 (lysosomal-associated membrane protein 1), SLC6A6 (solute carrier family 6 (neurotransmitter transporter, taurine) member 6), STX12 (syntaxin 12), APP (amyloid beta (A4) precursor protein) (all down) (Fig. 6A**; Table S11**). Comparing WT GFP-ATG5 against GFP-ATG5^K13^°^R^ surface proteomes revealed that no proteins were significantly increased, whereas those whose levels were reduced >1.3-fold included: LAMP1, BST2, SLC27A4, APP, LSS (lanosterol synthase), LMAN1 (lectin, mannose-binding 1), LRPAP1 (low density lipoprotein receptor-related protein associated protein 1) (Fig. 6B**; Table S11**). The absence of proteins increasing in this pairwise comparison, and the duplication of certain proteins whose surface levels decreased when comparing WT GFP-ATG5 with both GFP and GFP-ATG5^K13^°^R^ (namely, LAMP1, BST2, APP) (Fig. 6A**, B; Table S11**), is indicative of a general reduction of surface protein levels during starvation in autophagy-competent cells. Comparing GFP-ATG5^K13^°^R^ against GFP revealed the following proteins that significantly changed following starvation: LAMP1, HK1 (hexokinase 1), MTHFD1L (methylenetetrahydrofolate dehydrogenase (NADP+ dependent] 1-like), SACM1L (SAC1 suppressor of actin mutations 1-like (yeast)), STT3A (STT3, subunit of the oligosaccharyltransferase complex, homolog A (S. cerevisiae)) (all up); and MOB1B (MOB kinase activator 1B), CAMK2D (calcium/calmodulin dependent protein kinase II, delta), ERBB2 (erb-b2 receptor tyrosine kinase 2), ACACA (acetyl-Coenzyme A carboxylase alpha) (all down) (Fig. 6C**; Table S11**).

Finally, we analyzed the surface proteomes obtained from fed and starved WT GFP-ATG5, GFP-ATG5^K13^°^R^- and GFP-expressing *atg5^−/−^* MEFs to derive pairwise comparisons of protein level change during starvation. Comparing the WT GFP-ATG5 and GFP surface proteomes revealed relatively few proteins that changed >1.3-fold (starved/fed), these being: DSTN (destrin), PARK7 (Parkinson disease (autosomal recessive, early onset) 7), PLOD2 (procollogen lysine, 2-oxoglutarate 5-dioxygenase 2), GCLM (glutamate-cysteine ligase, modifier subunit) (all up); and PRNP (prion protein), LAMP1; SYPL (all down) (Fig. 6D**; Table S12**). Comparing WT GFP-ATG5 against GFP-ATG5^K13^°^R^ surface proteomes revealed the following that significantly changed during starvation: CARM1 (coactivator-associated arginine methyltransferase 1), MTAP (methylthioadenosine phosphorylase), GCN1 (GCN1 activator of EIF2AK4), TLN1 (talin 1), IQGAP1 (IQ motif containing GTPase activating protein 1) (all up); and PROC (protein C), RAB2A, SORCS2 (sortilin-related VPS10 domain containing receptor 2) (all down) (Fig. 6E**; Table S12**). Finally, only CYP20A1 (cytochrome P450, family 20, subfamily a, polypeptide 1) (up) and FASN (fatty acid synthase) (down) were found to differ between the 2 autophagy-deficient cell-lines, GFP-ATG5^K13^°^R^ and GFP (Fig. 6F**; Table S12**). Amongst these changes, LAMP1 was again found to be decreased when comparing WT GFP-ATG5 against GFP-expressing cells (Fig. 6D**; Table S12**). Analysis of the LAMP1 ratios under fed conditions showed very little differences between the different cell-lines (see **Table S12**), arguing that changes in its steady state localization only occur in response to altered cellular energy status, in the presence of an active autophagy system. LAMP1 has been implicated in the release of ATP during immunogenic cell death, via packaging into autolysosomes [73], and interestingly, mitoxantrone-induced immunogenic ATP release was found to depend on autophagy and the presence of ATG5 in this model.

### Functional characterization of LAT1 surface expression and autophagy competence

**Figure 7.**
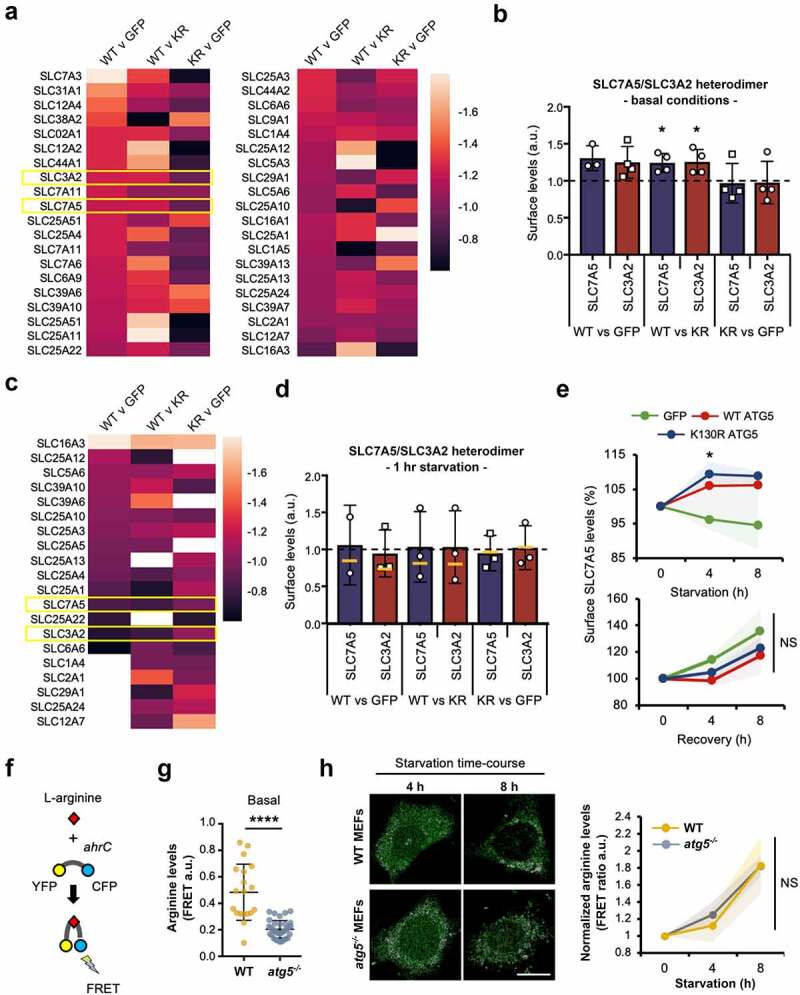
Surface amino acid transporter abundancies and basal cytosolic arginine levels are affected by autophagy capability. (**A**) Combinatorial heatmap analysis of selected surface transporter abundancies obtained from the SILAC datasets, compared between WT GFP-ATG5, GFP-ATG5^K13^°^R^ and GFP rescued *atg5^−/−^* MEFs (n = 3-4). SLC3A2 and SLC7A5 are highlighted in the boxed regions. (**B**) Basal surface SLC3A2-SLC7A5 levels are higher in cells expressing WT GFP-ATG5 (T-test; * = p<0.05). (**C**) Combinatorial heatmap analysis of selected surface transporters (n = 3). SLC3A2 and SLC7A5 are highlighted in the boxed regions. Where no color is shown, transporters were not identified in the relevant pairwise starved datasets. (**D**) SLC7A5-SLC3A2 surface levels normalise following a short period of starvation (1 h) across the different rescued *atg5*^−/−^ cell-lines. Bars show values after 1 h starvation; yellow lines indicate starvation-induced changes in relative abundance (starvation/basal). Data for SLC7A5 in the WT GFP-ATG5 vs. GFP pairwise analysis are depicted despite this transporter being identified in only 2 of the 3 SILAC datasets. (**E**) Extended starvation is associated with increased surface SLC7A5 levels in *atg5^−/−^* MEFs expressing either WT GFP-ATG5 or GFP-ATG5^K13^°^R^ relative to GFP expressing cells. Surface SLC7A5 levels were assessed by flow cytometry during starvation (up to 8 h; top) and following amino acid/growth factor replenishment (up to 8 h recovery; bottom). Graphs show means +/- SD (shaded areas). NS = not significant. (**F-H**) Arginine FRET suggests that autophagy capability and the presence of ATG5 influence cytosolic arginine levels. (**F**) Cartoon explaining the arginine FRET reporter, comprising YFP and CFP separated by the *ahrC* arginine repressor which undergoes a conformational change upon arginine binding to enable FRET. (**G**) Basal cytosolic arginine levels in wild-type and *atg5^−/−^* MEFs (a.u. = arbitrary units; **** = p<0.0001). (**H**) Changes in cytosolic arginine levels during prolonged starvation in wild-type and *atg5^−/−^* MEFs. Means +/- SD (shaded areas). NS = not significant.

As they contribute to the maintenance of homeostatic levels of solutes in proliferating cells, we were especially interested in how plasma membrane transport complexes (including the amino acid transporter family) were influenced by autophagy capability—compensatory enrichment of cell surface amino acid transporters might be predicted in autophagy-deficient cells. Variations in surface levels of transporter family proteins detected in at least 3 of the 4 repeat assays across all cell-lines are depicted as heat maps in Fig. 7A, and individual raw data sets are shown in **Table S6**. We focused our analysis on the L-type amino acid transporter, SLC7A5/LAT1 (solute carrier family 7 (cationic amino acid transporter, y+ system), member 5); a sodium-independent transporter of neutral branched chain amino acids. It forms a functional, covalent complex with SLC3A2/4F2hc/CD98 (solute carrier family 3 (activators of dibasic and neutral amino acid transport), member 2), allowing further validation of our quantitative surface proteomics approach as their pairwise levels should be very similar across all conditions (note: the component SLC7A7 [solute carrier family 7 (cationic amino acid transporter, y+ system), member 7] that also associates with SLC3A2 was not detected in our samples). Although this transporter never exceeded the arbitrary 1.3-fold difference threshold between cell-types, its individual components did differ consistently by >1.2-fold (Fig. 7B; **Table S6**). Contrary to our initial expectations, levels of surface SLC7A5-SLC3A2 were found to be higher in autophagy competent WT GFP-ATG5 MEFs under basal (fed) conditions (significantly enriched when compared with GFP-ATG5^K13^°^R^ MEFs; Fig. 7B). Correspondingly, there were marginal differences between the two autophagy-incompetent cell-lines (Fig. 7B).

We next assessed surface transporter levels in response to starvation (Fig. 7C). Fewer transporters are depicted in Fig. 7C in comparison to Fig. 7A, as only those detected in at least 2 pairwise comparisons, across 3/3 starvation surface proteomes are reported. Data for SLC7A5 in the WT GFP-ATG vs GFP dataset is included for consistency, despite this protein only being detected in 2/3 datasets (Fig. 7C, D). Mean values following starvation are shown as bars in Fig. 7D, with fold change depicted by yellow lines. These data show that during starvation, levels of the SLC7A5-SLC3A2 transporter equalise when comparing WT GFP-ATG5 MEFs with either GFP-ATG5^K13^°^R^ or GFP MEFs, suggesting that autophagy-deficient cells initially elevate surface levels of this important amino acid transporter relative to autophagy-competent cells, possibly to compensate for reduced catabolic amino acid mobilization via the autophagy system. These data should be viewed with some caution, however, due to the high variability between experiments. To expand on these findings, we therefore used flow cytometry as an orthogonal approach to measure changes in surface SLC7A5 (and by inference, SLC3A2) levels during prolonged starvation (up to 8 h). Intriguingly, these data suggested that the presence of ATG5 in either WT GFP-ATG5 or GFP-ATG5^K13^°^R^ formats supported increased SLC7A5 levels during longer starvation periods (4-8 h) relative to GFP expressing *atg5^−/−^* MEFs (Fig. 7E). We then followed a similar approach to test the relationships between ATG5 status and surface SLC7A5 during recovery from 4-h starvation (Fig. 7E). In this case, there were no differences in rates of recovery of surface SLC7A5 between the cell lines.

To begin to understand the significance of autophagy-linked differences in cellular amino acid levels in relation to transporter trafficking during starvation, we used a Förster resonance energy transfer (FRET) sensor [74] to measure cellular L-arginine levels. Arginine is a semi-essential amino acid, requiring dietary uptake to compensate for insufficient biosynthesis. As a source of nitric oxide, it plays important roles in cellular stress responses in health and disease, and crucially, arginine is a major player in lysosome-mediated nutrient sensing, mobilization and MTOR signalling (e.g. [75]). The arginine FRET sensor is based on the product of the *Bacillus subtilis ahrC* gene, an arginine repressor, and reports changes in cytosolic arginine levels in the low micromolar range (Fig. 7F) [74]. We used confocal microscopy to measure basal FRET levels and changes in FRET over a starvation time-course in wild-type and *atg5^−/−^* MEFs (Fig. 7F, G). In the basal (fed) state, arginine levels were significantly higher in wild-type cells, consistent with autophagy-deficient *atg5^−/−^* MEFs operating with lower cytosolic free arginine concentrations, perhaps as a consequence of the absence of basal autophagic long-lived protein turnover. During starvation, however, free arginine levels increased at comparable rates in wild-type and *atg5^−/−^* MEFs (Fig. 7H). This unexpected finding suggests that even in the absence of the canonical autophagy pathway, cells are able to mobilize important amino acids for anabolic processes through as yet undetermined routes.

In summary, our investigations have revealed strong interactions between autophagosome assembly factors—specifically ATG5 and the ATG12–ATG5 conjugate—and the clathrin-mediated trafficking machinery (Fig. 2). We provide evidence that PIK3C2A and HIP1R are candidate autophagy regulators that interact with ATG12–ATG5 (Fig. 3, 4). Quantitative analysis of the surface proteomes of the MEF cell-lines in fed and starved states revealed no obvious patterns in the classes of proteins whose surface levels differed between cell-lines, but a number of interesting proteins were identified whose plasma membrane distribution appeared to be influenced by autophagy capability and the availability of ATG5, including several solute transporters (Fig. 5, 6, 7). These data can form a framework for analysis of the influence of the core autophagy machinery on intracellular trafficking in basal and stressed conditions, particularly with reference to amino acid and other solute transporter families.

## Materials and Methods

### Reagents and antibodies

Unless stated otherwise, all reagents were from Sigma: GFP-Trap beads (Chromotek, GTA-100); BafA1 (Millipore, 475984) was used at 200 nM; DAPI (Life Technologies, D121490) was used at 100 ng/mL; puromycin (Sigma, P8833) was used at 2 µg/mL. Starvation was carried out either using Hanks’ Balanced Salt Solution (Thermo Fisher Scientific, 14025092), or with starvation media comprising 140 mM NaCl, 1 mM CaCl_2_, 1 mM MgCl_2_, 5 mM glucose, 20 mM HEPES, pH 7.4, supplemented with 1% (w:v) bovine serum albumin (BSA; Sigma, A0281) [5]. Cre recombinase lentiviral particles were a gift from Prof. James Uney (Bristol). Primary antibodies used were: anti-ATG5 (Sigma, A0856); anti-LC3B (Sigma, L8918); anti-LC3B (Cell Signaling Technology, 2956); anti-TUBA/β-tubulin (B-5-1-2; Sigma, T6074); anti-GFP (Covance, MMS-118P); anti-PIK3C2A (Cell Signaling Technology, 12402); anti-IGFR2/CI-M6PR (Abcam, ab2733); anti-SQSTM1/p62 (GeneTex, GT1478); anti-HIP1R (Abcam, ab226197); anti-ATG16L1 (MBL Life Sciences, M150-3); anti-ATG3 (MBL Life Sciences, M133-3); anti-CLTC (Sigma-Aldrich, C1860); anti-WIPI2 (AbD Serotech, MCA5780GA); anti-DNM1 (Abcam, ab13251); anti-DNM2 (Abcam, ab65556); anti-SNX9 (Santa Cruz Biotechnology, sc-166863); anti-SLC7A5/LAT1 Alexa Fluor 647-conjugate (R & D Systems, FAB10390R); anti-SLC27A4 (ABclonal, A16102). HRP-tagged secondary antibodies were from Stratech Scientific (G32-62G-SGC; G33-62G-SGC); fluorescent Alexa Fluor-tagged antibodies were from Thermo Fisher Scientific (A21202; A21206; A10037; A10042). pHRodo Red was from Thermo Fisher Scientific (P35372).

### cDNA and lentiviral constructs

Wild-type and *atg5^K13^°^R^* mouse cDNA (synthesized by Eurofins Genomics) was sub-cloned into pLVX-puro (Takara, 632164) using Afe1 and BamHI restriction sites. Lentiviruses were generated in HEK293T cells (ECACC, 12022001) by transfection with cDNAs along with packaging vectors pMD2.G and pAX2 (a gift from Prof. Pete Cullen, Bristol). The FRET-based arginine sensor cDNA was a kind gift of Francois Verrey (Institute pf Physiology, Zurich, Switzerland) [74]. This sensor was genetically engineered from the *ahrC* gene, a repressor/activator of arginine synthesis in *Bacillus subtilis*, cloned between FCP and YFP reporters under the control of the CMV promoter,

### Cell-lines and cell culture

Unless otherwise stated, cells were grown in DMEM containing 4500 mg/mL glucose (Sigma, D5796) at 37°C with 5% CO_2_. The following cell-lines were used: *atg5* wild-type and *atg5* null MEFs, and *atg3* wild-type and *atg3*-null MEFs (from Dr Sharon Tooze, CRICK Institute, London); *pik3c2a^fl^°^x/fl^°^x^* MEFs [58]; *hip1r* null MEFs (from Dr Theodora Ross, UT Southwestern Medical Center, USA, [76]; HEK293T (ATCC, CRL-3216). *atg5*- and *atg3*-null MEFs stably expressing GFP, WT GFP-ATG5 or GFP-ATG5^K13^°^R^ were produced by lentiviral transduction. Lentiviral particles were collected at 48 h, cleared by centrifugation at 2900*g*, 10 min, then passed through a 0.45-µm polyethersulfone filter (Corning, CLS431220) to be used immediately or stored at -80°C. MEFs were transduced on 6-cm plates at ~40% confluency by two rounds of viral addition. Cells were selected using puromycin.

### GFP-Trap

For GFP-Trap immunoisolation, cells in 10-cm plates were washed twice with ice cold PBS (Thermo Fisher Scientific, 10010-023), and 1 ml of lysis buffer (10 mM Tris base, 150 mM NaCl, 0.5% [v:v] octylphenoxypolyethoxyethanol [Igepal CA-630, Sigma, I8896], 1 mM PMSF [Sigma, P7626], 2 mM MgCl_2_, protease inhibitors [Roche, 11697498001], pH 7.5) was added. Cells were scraped and lysates collected and incubated on ice for 20 min. Lysates were cleared by centrifugation at 20000*g* for 15 min at 4^°^C, and added to pre-equilibrated beads and rotated for 1 h at 4^°^C. The sample was then spun at 600*g* for 2 min at 4^°^C to pellet beads, which were washed 3x in wash buffer (10 mM Tris base; 150 mM NaCl; 1 mM PMSF; 2 mM MgCl_2_; protease inhibitors) before they were resuspended in SDS-PAGE gel sample buffer.

### Surface biotinylation

Cells were grown to confluency in 15-cm dishes (for SILAC) or 6-well plates (for immunoblotting analysis). Sulfo-NHS-SS Biotin (Thermo Fisher Scientific, A39258) was prepared at 0.2 mg/mL in ice-cold PBS, and cells were incubated with 5 mL/1 mL PBS-biotin on ice for 30 min. Cells were quenched for 10 min (50 mM Tris, 100 mM NaCl, pH 7.5), and then lysed in 1 mL/200 µl lysis buffer (PBS with 2% Triton X-100 [Sigma, 9036-19-5] and protease inhibitors). Cells were scraped and the lysates cleared at 20000*g* for 10 min at 4^°^C. The cleared lysates were then incubated with Streptavidin Sepharose (GE Healthcare, 17511301) for 30 min at 4^°^C under rotation. After extensive washes, samples were resuspended in SDS-PAGE gel sample buffer.

### Quantitative SILAC proteomics

For SILAC, cells were grown for 8 doublings in DMEM for SILAC (Thermo Fisher Scientific, 89985), containing 4500 mg/mL glucose and 4 mM L-glutamine, supplemented with 100 µg/mL penicillin, 100 µg/mL streptomycin and 10% fetal bovine serum (Sigma, F0392). The following amino acids were added: “light” medium (R0/K0), Arg0 (Sigma, A6969) Lys0 (Sigma, L8662); “medium” medium (R6/K4), Arg6 (Sigma, 643440) Lys4 (Thermo, 88437); “heavy” medium (R10/K8), Arg10 (Silantes, 201604301) Lys8 (Silantes, 211204302). Liquid chromatography coupled to tandem mass spectrometry (LC-MS/MS) analysis was carried out using an LTQ Orbitrap Velos mass spectrometer (Thermo Fisher Scientific) in the Bristol University Proteomics facility. Gels were cut into 6 slices using an automated digestion unit (ProGest; Digilab UK), and a trypsin digest performed followed by peptide fractionation [nano-HPLC system (UltiMate 3000, Dionex)], ionization (ES542; Proeson) and MS/MS. MS data was acquired using Xcalibar v2.1 software (Thermo Fisher Scientific). The results were first analyzed using a Sequest search against the Uniprot mouse database and then filtered to remove low confidence peptides [<5% false-discovery rate (FDR)]. Using Excel, proteins with a unique peptide number <2 were discarded. The unique peptide number displays the number of peptide sequences unique to a protein group. STRING analysis (Search Tool for the Retrieval of Interacting Genes/Proteins; http://string-db.org/) was used to identify putative protein interaction networks.

### Microscopy

Fixed and live-cell images were obtained using an Olympus IX-71 inverted microscope (60x Uplan Fluorite objective; 0.65-1.25 NA, oil immersion lens) fitted with a CoolSNAP HQ CCD camera (Photometrics, AZ) driven by MetaMorph software (Molecular Devices). MetaMorph software was used to quantify puncta numbers. A TopHat morphology filter was used to score circular objects of 5 pixels (~1 µm) diameter. An automated cell count was then performed to count the number of selected items. For a typical experiment, ten random fields were imaged and puncta numbers per cell in each field was counted. This was repeated three times. All other image analysis was carried out using Fiji. Confocal microscopy was carried out using a Leica SP5-AOBS confocal laser scanning microscope (63x oil immersion objective, 1.4NA; or 100x oil immersion objective, 1.4NA) attached to a Leica DM I6000 inverted epifluorescence microscope. Laser lines were: 100mW Argon (for 458, 488, 514 nm excitation); 2mW Orange HeNe (594 nm); and 50mW diode laser (405 nm). The microscope was run using Leica LAS AF software (Leica, Germany). FRET analysis was also carried out on this microscope.

For CLEM, cells were grown on gridded 3-cm imaging dishes (MatTek, P35G-1.5-14-CGR), imaged live by widefield fluorescence imaging to observe GFP-ATG5 puncta under starvation conditions, and fixed by adding glutaraldehyde to 3%. Cells were processed for transmission electron microscopy as follows: cells were washed in 0.1 M sodium cacodylate, osmicated for 1 h (1% osmium tetroxide in 0.1 M sodium cacodylate), dehydrated by increasing ethanol concentration steps, then embedded in TAAB 812 resin (TAAB, T022). Blocks were then trimmed to the cells of interest, sectioned, then imaged using a FEI Technai 12 120kV transmission electron microscope. Images were captured using a FEI Ceta 4k x4k CCD camera.

### Flow cytometry

Flow cytometry was carried out to assess cell surface levels of LAT1. 100,000 cells were harvested using EDTA and cleared at 2700 x g for 3 min. The pellet was resuspended in 30 µl PBS with 2% vol:vol FBS including anti-SLC7A5/LAT1-647 primary antibody (1:50, v:v), and incubated for 30 min on ice. A single wash with PBS with 2% vol:vol FBS was then carried out. The cells were then resuspended in PBS containing 2% v:v FBS and 1% w:v PFA. Samples were analyzed in the flow cytometry facility at Bristol University (under the guidance of Dr Andrew Hermann) using a cell analyzer BD FACSCANTO II (BD Biosciences). FlowJo software was used to analyze the data.

### Statistical analysis

For the analysis of imaging and densitometry data, one-way ANOVA calculations with Tukey’s multiple comparison tests were carried out using GraphPad Prism. For the surface interactomes, data were normalized to account for uneven incorporation of the SILAC labelled media as follows: the median intensity value for each condition was subtracted from each individual protein intensity value to create a new median of 1. Log_2_ of each intensity value was calculated, and a T-test was then carried out for all proteins present within at least 3 of the datasets against the median. Proteins having a fold-difference between pairwise comparisons of >1.3 and p value <0.05 were considered of interest. For the starved datasets, a second analysis in which the fold change within pairwise samples was compared to the fed state was carried out (starved/fed), with fold-difference >1.3 and p value <0.5 signifying interest.

## Supplementary Material

Supplemental Material

## Data Availability

The authors confirm that the data supporting the findings of this study are available within the article [and/or] its supplementary materials.

## References

[1] Dikic, I. and Z. Elazar, Mechanism and medical implications of mammalian autophagy. Nat Rev Mol Cell Biol, 2018. 19(6): p. 349–364. 29618831 10.1038/s41580-018-0003-4

[2] Nakatogawa, H., Two ubiquitin-like conjugation systems that mediate membrane formation during autophagy. Essays Biochem, 2013. 55: p. 39–50. 24070470 10.1042/bse0550039

[3] Lamb, C.A., T. Yoshimori, and S.A. Tooze, The autophagosome: origins unknown, biogenesis complex. Nat Rev Mol Cell Biol, 2013. 14(12): p. 759–74. 24201109 10.1038/nrm3696

[4] Valverde, D.P., et al., *ATG2 transports lipids to promote autophagosome biogenesis*. J Cell Biol, 2019. 10.1083/jcb.201811139 PMC6548141 30952800

[5] Axe, E.L., et al., Autophagosome formation from membrane compartments enriched in phosphatidylinositol 3-phosphate and dynamically connected to the endoplasmic reticulum. J Cell Biol, 2008. 182(4): p. 685–701. 18725538 10.1083/jcb.200803137 PMC2518708

[6] Ge, L., et al., Remodeling of ER-exit sites initiates a membrane supply pathway for autophagosome biogenesis. EMBO Rep, 2017. 18(9): p. 1586–1603. 28754694 10.15252/embr.201744559 PMC5579361

[7] Ravikumar, B., et al., Plasma membrane contributes to the formation of pre-autophagosomal structures. Nat Cell Biol, 2010. 12(8): p. 747–57. 20639872 10.1038/ncb2078 PMC2923063

[8] Hailey, D.W., et al., Mitochondria supply membranes for autophagosome biogenesis during starvation. Cell, 2010. 141(4): p. 656–67. 20478256 10.1016/j.cell.2010.04.009 PMC3059894

[9] Shima, T., H. Kirisako, and H. Nakatogawa, *COPII vesicles contribute to autophagosomal membranes*. J Cell Biol, 2019. 10.1083/jcb.201809032 PMC6504894 30787039

[10] Puri, C., et al., The RAB11A-Positive Compartment Is a Primary Platform for Autophagosome Assembly Mediated by WIPI2 Recognition of PI3P-RAB11A. Dev Cell, 2018. 45(1): p. 114–131 e8. 29634932 10.1016/j.devcel.2018.03.008 PMC5896254

[11] Behrends, C., et al., Network organization of the human autophagy system. Nature, 2010. 466(7302): p. 68–76. 20562859 10.1038/nature09204 PMC2901998

[12] Kern, A., I. Dikic, and C. Behl, The integration of autophagy and cellular trafficking pathways via RAB GAPs. Autophagy, 2015. 11(12): p. 2393–7. 26565612 10.1080/15548627.2015.1110668 PMC4835203

[13] Carroll, B., et al., The TBC/RabGAP Armus coordinates Rac1 and Rab7 functions during autophagy. Dev Cell, 2013. 25(1): p. 15–28. 23562278 10.1016/j.devcel.2013.03.005 PMC3898768

[14] Longatti, A., et al., TBC1D14 regulates autophagosome formation via Rab11- and ULK1-positive recycling endosomes. J Cell Biol, 2012. 197(5): p. 659–75. 22613832 10.1083/jcb.201111079 PMC3365497

[15] Lamb, C.A., et al., TBC1D14 regulates autophagy via the TRAPP complex and ATG9 traffic. EMBO J, 2016. 35(3): p. 281–301. 26711178 10.15252/embj.201592695 PMC4741301

[16] Itoh, T., et al., OATL1, a novel autophagosome-resident Rab33B-GAP, *regulates autophagosomal maturation*. J Cell Biol, 2011. 192(5): p. 839–53. 21383079 10.1083/jcb.201008107 PMC3051816

[17] Popovic, D. and I. Dikic, TBC1D5 and the AP2 complex regulate ATG9 trafficking and initiation of autophagy. EMBO Rep, 2014. 15(4): p. 392–401. 24603492 10.1002/embr.201337995 PMC3989670

[18] Puri, C., et al., Diverse autophagosome membrane sources coalesce in recycling endosomes. Cell, 2013. 154(6): p. 1285–99. 24034251 10.1016/j.cell.2013.08.044 PMC3791395

[19] Anton, Z., et al., A heterodimeric SNX4–SNX7 SNX-BAR autophagy complex coordinates ATG9A trafficking for efficient autophagosome assembly. J Cell Sci, 2020. **133**(14). 10.1242/jcs.246306 PMC7375690 32513819

[20] Knaevelsrud, H., et al., Membrane remodeling by the PX-BAR protein SNX18 promotes autophagosome formation. J Cell Biol, 2013. 202(2): p. 331–49. 23878278 10.1083/jcb.201205129 PMC3718966

[21] Soreng, K., et al., SNX18 regulates ATG9A trafficking from recycling endosomes by recruiting Dynamin-2. EMBO Rep, 2018. **19**(4). 10.15252/embr.201744837 PMC5891424 29437695

[22] Judith, D., et al., ATG9A shapes the forming autophagosome through Arfaptin 2 and phosphatidylinositol 4-kinase IIIbeta. J Cell Biol, 2019. 218(5): p. 1634–1652. 30917996 10.1083/jcb.201901115 PMC6504893

[23] Noda, T., Autophagy in the context of the cellular membrane-trafficking system: the enigma of Atg9 vesicles. Biochem Soc Trans, 2017. 45(6): p. 1323–1331. 29150528 10.1042/BST20170128 PMC5730941

[24] Tooze, S.A., A. Abada, and Z. Elazar, Endocytosis and autophagy: exploitation or cooperation? Cold Spring Harb Perspect Biol, 2014. 6(5): p. a018358. 24789822 10.1101/cshperspect.a018358 PMC3996471

[25] Cadwell, K. and J. Debnath, Beyond self-eating: The control of nonautophagic functions and signaling pathways by autophagy-related proteins. J Cell Biol, 2018. 217(3): p. 813–822. 29237720 10.1083/jcb.201706157 PMC5839790

[26] Malhotra, V., Unconventional protein secretion: an evolving mechanism. EMBO J, 2013. 32(12): p. 1660–4. 23665917 10.1038/emboj.2013.104 PMC3680731

[27] Rabouille, C., V. Malhotra, and W. Nickel, Diversity in unconventional protein secretion. J Cell Sci, 2012. **125**(Pt 22): p. 5251–5. 23377655 10.1242/jcs.103630

[28] Dupont, N., et al., Autophagy-based unconventional secretory pathway for extracellular delivery of IL-1beta. EMBO J, 2011. 30(23): p. 4701–11. 22068051 10.1038/emboj.2011.398 PMC3243609

[29] Gee, H.Y., et al., Rescue of DeltaF508-CFTR trafficking via a GRASP-dependent unconventional secretion pathway. Cell, 2011. 146(5): p. 746–60. 21884936 10.1016/j.cell.2011.07.021

[30] Gump, J.M., et al., Autophagy variation within a cell population determines cell fate through selective degradation of Fap-1. Nat Cell Biol, 2014. 16(1): p. 47–54. 24316673 10.1038/ncb2886 PMC3876036

[31] Roy, S., et al., Autophagy-Dependent Shuttling of TBC1D5 Controls Plasma Membrane Translocation of GLUT1 and Glucose Uptake. Mol Cell, 2017. 67(1): p. 84–95 e5. 28602638 10.1016/j.molcel.2017.05.020 PMC5522182

[32] Fraser, J., et al., Targeting of early endosomes by autophagy facilitates EGFR recycling and signalling. EMBO Rep, 2019. 20(10): p. e47734. 31448519 10.15252/embr.201947734 PMC6776898

[33] Lee, H.K., et al., In vivo requirement for Atg5 in antigen presentation by dendritic cells. Immunity, 2010. 32(2): p. 227–39. 20171125 10.1016/j.immuni.2009.12.006 PMC2996467

[34] Schmid, D. and C. Munz, Innate and adaptive immunity through autophagy. Immunity, 2007. 27(1): p. 11–21. 17663981 10.1016/j.immuni.2007.07.004 PMC7118777

[35] Loi, M., et al., Macroautophagy Proteins Control MHC Class I Levels on Dendritic Cells and Shape Anti-viral CD8(+) T Cell Responses. Cell Rep, 2016. 15(5): p. 1076–1087. 27117419 10.1016/j.celrep.2016.04.002

[36] Kimura, T., et al., Dedicated SNAREs and specialized TRIM cargo receptors mediate secretory autophagy. EMBO J, 2017. 36(1): p. 42–60. 27932448 10.15252/embj.201695081 PMC5210154

[37] Heckmann, B.L., et al., *LC3-Associated Endocytosis Facilitates beta-Amyloid Clearance and Mitigates Neurodegeneration in Murine Alzheimer’s Disease*. Cell, 2019. 10.1016/j.cell.2019.05.056 PMC6689199 31257024

[38] Mizushima, N., et al., Dissection of autophagosome formation using Apg5-deficient mouse embryonic stem cells. J Cell Biol, 2001. 152(4): p. 657–68. 11266458 10.1083/jcb.152.4.657 PMC2195787

[39] Tsuboyama, K., et al., The ATG conjugation systems are important for degradation of the inner autophagosomal membrane. Science, 2016. 354(6315): p. 1036–1041. 27885029 10.1126/science.aaf6136

[40] Hayashi-Nishino, M., et al., A subdomain of the endoplasmic reticulum forms a cradle for autophagosome formation. Nat Cell Biol, 2009. 11(12): p. 1433–7. 19898463 10.1038/ncb1991

[41] Ktistakis, N.T., E. Karanasios, and M. Manifava, Dynamics of autophagosome formation: a pulse and a sequence of waves. Biochem Soc Trans, 2014. 42(5): p. 1389–95. 25233420 10.1042/BST20140183

[42] Ktistakis, N.T. and S.A. Tooze, Digesting the Expanding Mechanisms of Autophagy. Trends Cell Biol, 2016. 26(8): p. 624–35. 27050762 10.1016/j.tcb.2016.03.006

[43] Chen, D., et al., A mammalian autophagosome maturation mechanism mediated by TECPR1 and the Atg12-Atg5 conjugate. Mol Cell, 2012. 45(5): p. 629–41. 22342342 10.1016/j.molcel.2011.12.036 PMC3299828

[44] Otomo, C., et al., Structure of the human ATG12~ATG5 conjugate required for LC3 lipidation in autophagy. Nat Struct Mol Biol, 2013. 20(1): p. 59–66. 23202584 10.1038/nsmb.2431 PMC3540207

[45] Romanov, J., et al., Mechanism and functions of membrane binding by the Atg5-Atg12/Atg16 complex during autophagosome formation. EMBO J, 2012. 31(22): p. 4304–17. 23064152 10.1038/emboj.2012.278 PMC3501226

[46] Hooper, K.M., et al., *V-ATPase is a universal regulator of LC3 associated phagocytosis and non-canonical autophagy*. bioRxiv, 2021. https://www.biorxiv.org/content/10.1101/2021.05.20.444917v1.

[47] Xu, Y., et al., A Bacterial Effector Reveals the V-ATPase-ATG16L1 Axis that Initiates Xenophagy. Cell, 2019. 178(3): p. 552–566 e20. 31327526 10.1016/j.cell.2019.06.007

[48] Ishibashi, K., et al., Atg16L2, a novel isoform of mammalian Atg16L that is not essential for canonical autophagy despite forming an Atg12-5-16L2 complex. Autophagy, 2011. 7(12): p. 1500–13. 22082872 10.4161/auto.7.12.18025 PMC3288023

[49] Kaksonen, M. and A. Roux, Mechanisms of clathrin-mediated endocytosis. Nat Rev Mol Cell Biol, 2018. 19(5): p. 313–326. 29410531 10.1038/nrm.2017.132

[50] Paczkowski, J.E., B.C. Richardson, and J.C. Fromme, Cargo adaptors: structures illuminate mechanisms regulating vesicle biogenesis. Trends Cell Biol, 2015. 25(7): p. 408–16. 25795254 10.1016/j.tcb.2015.02.005 PMC4475447

[51] Sou, Y.S., et al., The Atg8 conjugation system is indispensable for proper development of autophagic isolation membranes in mice. Mol Biol Cell, 2008. 19(11): p. 4762–75. 18768753 10.1091/mbc.E08-03-0309 PMC2575156

[52] Devereaux, K., et al., Regulation of mammalian autophagy by class II and III PI 3-kinases through PI3P synthesis. PLoS One, 2013. 8(10): p. e76405. 24098492 10.1371/journal.pone.0076405 PMC3789715

[53] Falasca, M. and T. Maffucci, Regulation and cellular functions of class II phosphoinositide 3-kinases. Biochem J, 2012. 443(3): p. 587–601. 22507127 10.1042/BJ20120008

[54] Jean, S. and A.A. Kiger, Coordination between RAB GTPase and phosphoinositide regulation and functions. Nat Rev Mol Cell Biol, 2012. 13(7): p. 463–70. 22722608 10.1038/nrm3379

[55] Backer, J.M., The regulation and function of Class III PI3Ks: novel roles for Vps34. Biochem J, 2008. 410(1): p. 1–17. 18215151 10.1042/BJ20071427

[56] Posor, Y., et al., Spatiotemporal control of endocytosis by phosphatidylinositol-3,4-bisphosphate. Nature, 2013. 499(7457): p. 233–7. 23823722 10.1038/nature12360

[57] McMahon, H.T. and E. Boucrot, Molecular mechanism and physiological functions of clathrin-mediated endocytosis. Nat Rev Mol Cell Biol, 2011. 12(8): p. 517–33. 21779028 10.1038/nrm3151

[58] Yoshioka, K., et al., Endothelial PI3K-C2alpha, a class II PI3K, *has an essential role in angiogenesis and vascular barrier function*. Nat Med, 2012. 18(10): p. 1560–9. 22983395 10.1038/nm.2928

[59] Tiosano, D., et al., Mutations in PIK3C2A cause syndromic short stature, skeletal abnormalities, *and cataracts associated with ciliary dysfunction*. PLoS Genet, 2019. 15(4): p. e1008088. 31034465 10.1371/journal.pgen.1008088 PMC6508738

[60] Franco, I., et al., Phosphoinositide 3-Kinase-C2alpha Regulates Polycystin-2 Ciliary Entry and Protects against Kidney Cyst Formation. J Am Soc Nephrol, 2016. 27(4): p. 1135–44. 26271513 10.1681/ASN.2014100967 PMC4814170

[61] Engqvist-Goldstein, A.E., et al., An actin-binding protein of the Sla2/Huntingtin interacting protein 1 family is a novel component of clathrin-coated pits and vesicles. J Cell Biol, 1999. 147(7): p. 1503–18. 10613908 10.1083/jcb.147.7.1503 PMC2174256

[62] Engqvist-Goldstein, A.E., et al., The actin-binding protein Hip1R associates with clathrin during early stages of endocytosis and promotes clathrin assembly in vitro. J Cell Biol, 2001. 154(6): p. 1209–23. 11564758 10.1083/jcb.200106089 PMC2150824

[63] Poupon, V., et al., Clathrin light chains function in mannose phosphate receptor trafficking via regulation of actin assembly. Proc Natl Acad Sci U S A, 2008. 105(1): p. 168–73. 18165318 10.1073/pnas.0707269105 PMC2224180

[64] Chen, C.Y. and F.M. Brodsky, Huntingtin-interacting protein 1 (Hip1) and Hip1-related protein (Hip1R) bind the conserved sequence of clathrin light chains and thereby influence clathrin assembly in vitro and actin distribution in vivo. J Biol Chem, 2005. 280(7): p. 6109–17. 15533940 10.1074/jbc.M408454200

[65] Legendre-Guillemin, V., et al., HIP1 and HIP12 display differential binding to F-actin, AP2, *and clathrin. Identification of a novel interaction with clathrin light chain*. J Biol Chem, 2002. 277(22): p. 19897–904. 11889126 10.1074/jbc.M112310200

[66] Legendre-Guillemin, V., et al., Huntingtin interacting protein 1 (HIP1) regulates clathrin assembly through direct binding to the regulatory region of the clathrin light chain. J Biol Chem, 2005. 280(7): p. 6101–8. 15533941 10.1074/jbc.M408430200

[67] Hyun, T.S., et al., HIP1 and HIP1r stabilize receptor tyrosine kinases and bind 3-phosphoinositides via epsin N-terminal homology domains. J Biol Chem, 2004. 279(14): p. 14294–306. 14732715 10.1074/jbc.M312645200

[68] Legendre-Guillemin, V., et al., ENTH/ANTH proteins and clathrin-mediated membrane budding. J Cell Sci, 2004. **117**(Pt 1): p. 9–18. 14657269 10.1242/jcs.00928

[69] Carreno, S., et al., Actin dynamics coupled to clathrin-coated vesicle formation at the trans-Golgi network. J Cell Biol, 2004. 165(6): p. 781–8. 15210728 10.1083/jcb.200403120 PMC2172402

[70] Coutts, A.S. and N.B. La Thangue, Actin nucleation by WH2 domains at the autophagosome. Nat Commun, 2015. 6: p. 7888. 26223951 10.1038/ncomms8888 PMC4532831

[71] Gimeno, R.E., Fatty acid transport proteins. Curr Opin Lipidol, 2007. 18(3): p. 271–6. 17495600 10.1097/MOL.0b013e3281338558

[72] Wu, S., et al., SLC27A4 regulate ATG4B activity and control reactions to chemotherapeutics-induced autophagy in human lung cancer cells. Tumour Biol, 2016. 37(5): p. 6943–52. 26662804 10.1007/s13277-015-4587-4

[73] Martins, I., et al., Molecular mechanisms of ATP secretion during immunogenic cell death. Cell Death Differ, 2014. 21(1): p. 79–91. 23852373 10.1038/cdd.2013.75 PMC3857631

[74] Vanoaica, L., et al., Real-time functional characterization of cationic amino acid transporters using a new FRET sensor. Pflugers Arch, 2016. 468(4): p. 563–72. 26555760 10.1007/s00424-015-1754-9

[75] Wyant, G.A., et al., mTORC1 Activator SLC38A9 Is Required to Efflux Essential Amino Acids from Lysosomes and Use Protein as a Nutrient. Cell, 2017. 171(3): p. 642–654 e12. 29053970 10.1016/j.cell.2017.09.046 PMC5704964

[76] Hyun, T.S., et al., Hip1-related mutant mice grow and develop normally but have accelerated spinal abnormalities and dwarfism in the absence of HIP1. Mol Cell Biol, 2004. 24(10): p. 4329–40. 15121852 10.1128/MCB.24.10.4329-4340.2004 PMC400480

