# Supplementary material for: The ATG5 interactome links clathrin-mediated vesicular trafficking with the autophagosome assembly machinery
Source: Autophagy Rep. 2022 Apr 7;1(1):88–118. doi: 10.1080/27694127.2022.2042054 (PMC9015699; doi:10.1080/27694127.2022.2042054)
Supplement: Supplemental Material [file KAUO_A_2042054_SM3247.zip › Supplementary information/Baines et al Supplemental Figures.docx]

**SUPPLEMENTAL FIGURES**

**Figure S1.** *atg5* null MEFs are autophagy deficient. Analysis of autophagy responses in wild-type and *atg5* null MEFs by (**A**) immunoblotting and (**B-D**) immunofluorescence. Cells were starved for 1 h in the absence or presence of BafA1. Refeeding was for 1 h in full-nutrient medium. Example immunofluorescence images (**B**) and quantification of WIPI2 (**C**) and LC3B (**D**) puncta are shown. Data show mean ± SEM; n = 3; 10 fields of cells per condition, per experiment. One-way ANOVA with post-hoc Tukey’s test: ** p < 0.01; *** p < 0.001. Bar: 10 µm.

**Figure S2.** SILAC-based proteomics analysis of interactions within the GFP-ATG5^K130R^ dataset. STRING analysis of interactions within the high confidence GFP-ATG5^K130R^ interactome, showing autophagy-related proteins and a second network comprising extracellular matrix components.

**Figure S3.** SILAC-based proteomics analysis of GFP-ATG5 interactors in the *atg3*^-/-^ MEF background. (**A**) STRING analysis of WT GFP-ATG5 interactors in the *atg3*^-/-^ MEF background. (**B**) STRING analysis of GFP-ATG5^K130R^ interactors in the *atg3*^-/-^ MEF background. (**C**) Immunoblot of GFP-TRAP affinity isolates in *atg3*^-/-^ MEFs stably expressing GFP, WT GFP-ATG5 or GFP-ATG5^K130R^ showing PIK3C2A and ATG5 immunoreactivity.

**Figure S4.** Analysis of endocytic transport in *atg5*^-/-^ MEFs rescued with ATG5 constructs. (**A**) Time-dependent uptake of transferrin Alexa Fluor 647 in *atg5*^-/-^ MEFs rescued with GFP, WT GFP-ATG5 or GFP-ATG5^K130R^; mean +/- SD. NS = non-significant. (**B-E**) Rescued *atg5*^-/-^ MEFs were incubated with pHrodo for the indicated times under basal conditions (**B, C**) or following 1 h of starvation, and then during the indicated times (**D, E**). The cells were imaged live on a confocal microscope and fluorescence intensity (MFI) was measured. Mean ± SD normalized against GFP; n = 4 (**C**) and n = 3 (**E**) independent experiments, measuring 10 cells per experiment. GFP vs. WT GFP-ATG5: * = p<0.05; ** = p< 0.01; *** = p<0.001. GFP vs. GFP-ATG5^K130R^: $ = p<0.05; $$ = p< 0.01; $$$ = p<0.001; WT GFP-ATG5 vs. K130R GFP-ATG5 # = p<0.05; ## = p< 0.01; ### = p<0.001.
