# Supplementary material for: The ATG5 interactome links clathrin-mediated vesicular trafficking with the autophagosome assembly machinery
Source: Autophagy Rep. 2022 Apr 7;1(1):88–118. doi: 10.1080/27694127.2022.2042054 (PMC9015699; doi:10.1080/27694127.2022.2042054)
Supplement: Supplemental Material [file KAUO_A_2042054_SM3247.zip › Supplementary information/Table S11.docx]

**Table S11.** Surface interactome in starved MEF rescue backgrounds.

| **WT GFP-ATG5 vs. GFP** | | | | | | | |
| --- | --- | --- | --- | --- | --- | --- | --- |
| ***Increased*** | | | | ***Decreased*** | | | |
| **Accession** | **Description** | **Mean** | ***P*-value** | **Accession** | **Description** | **Mean** | ***P*-value** |
| Q3V3R1 | MTHFD1L | 1.506 | 0.037687061 | E9PX70 | COL12A1 | 0.401 | 0.016454101 |
| G5E843 | ROBO1 | 1.483 | 0.025731268 | Q8R2Q8 | BST2 | 0.469 | 0.02475123 |
| A2A813 | PARK7 | 1.417 | 0.01063417 | Q8CE18 | Uncharacterised protein | 0.564 | 0.013425909 |
| Q8R464 | CADM4 | 1.370 | 0.049862472 | Q3TVX7 | SYPL | 0.62 | 0.031614541 |
| Q9R0B9 | PLOD2 | 1.340 | 0.047553399 | Q9CQW9 | IFITM3 | 0.63 | 0.046282885 |
| F7BWT7 | TSPAN15 | 1.307 | 0.003948324 | O78207 | H2-D1 | 0.632 | 0.041047422 |
| Q3THB3 | HNRNPM | 1.304 | 0.018914629 | Q3TWF3 | APP | 0.65 | 0.043893734 |
|  |  |  |  | G3UXZ5 | PSME1 | 0.663 | 0.042184701 |
|  |  |  |  | P11438 | LAMP1 | 0.665 | 0.032615735 |
|  |  |  |  | O35316 | SLC6A6 | 0.674 | 0.037689164 |
|  |  |  |  | Q3TDG9 | STX12 | 0.758 | 0.01266362 |
|  | | | | | | | |
| **WT GFP-ATG5 vs. K130R GFP-ATG5** | | | | | | | |
| ***Decreased*** | | | | | | | |
| **Accession** | **Description** | **Mean** | ***P*-value** | **Accession** | **Description** | **Mean** | ***P*-value** |
| P11438 | LAMP1 | 0.389 | 0.00064169 | P24369 | PPIB | 0.690 | 0.043396898 |
| Q8R2Q8 | BST2 | 0.489 | 0.018887447 | Q9R1R9 | RDH11 | 0.699 | 0.014634982 |
| Q8BLN5 | LSS | 0.539 | 0.015667318 | Q9D0F3 | LMAN1 | 0.701 | 0.022609471 |
| Q3TWF3 | APP | 0.632 | 0.000819975 | Q6ZQI3 | MLEC | 0.709 | 0.04219749 |
| Q61398 | PCOLCE | 0.637 | 0.030960639 | Q80UU9 | PGRMC2 | 0.723 | 0.039208203 |
| E9QMJ5 | ADGRE5 | 0.645 | 0.04195319 | Q05DV1 | POR | 0.733 | 0.001021338 |
| Q6PB52 | LRPAP1 | 0.646 | 0.036486923 | Q61833 | RPN2 | 0.744 | 0.00793402 |
| Q91VE0 | SLC27A4 | 0.648 | 0.011679863 | Q08879 | FBLN1 | 0.746 | 0.044594835 |
| P27773 | PDIA3 | 0.658 | 0.049089537 | O70503 | HSD17B12 | 0.762 | 0.005339892 |
| Q9QXT0 | CNPY2 | 0.660 | 0.038651952 | O08807 | PRDX4 | 0.767 | 0.035518398 |
| Q3TDN2 | FAF2 | 0.669 | 0.011108781 | Q5NCU4 | SPARC | 0.767 | 0.026942681 |
|  | | | | | | | |
| **K130R GFP-ATG5 vs. GFP** | | | | | | | |
| ***Increased*** | | | | ***Decreased*** | | | |
| **Accession** | **Description** | **Mean** | ***P*-value** | **Accession** | **Description** | **Mean** | ***P*-value** |
| P11438 | LAMP1 | 1.920 | 0.025481169 | P11438 | MOB1B | 0.660 | 0.006370983 |
| Q9EP69 | SAC1 | 1.783 | 0.0451057 | Q9EP69 | PPIC | 0.674 | 0.049442382 |
| G3UVV4 | HK1 | 1.685 | 0.001635713 | G3UVV4 | RPL7 | 0.716 | 0.024964515 |
| Q3V3R1 | MTHFD1L | 1.666 | 0.012400546 | Q3V3R1 | RPL28 | 0.718 | 0.012843299 |
| A1L2Z3 | EMC1 | 1.526 | 0.044983442 | A1L2Z3 | CAMK2D | 0.746 | 0.014498686 |
| P46978 | STT3A | 1.471 | 0.006492825 | P46978 | ACACA | 0.750 | 0.027215708 |
| Q8BVQ5 | PPME1 | 1.442 | 0.021765395 | Q8BVQ5 | ERBB2 | 0.758 | 0.005412206 |
| Q8BKE6 | CYP20A1 | 1.438 | 0.023491593 | Q8BKE6 | IL1RAP | 0.758 | 0.014312666 |
| P58242 | SMPDL3B | 1.400 | 0.023246228 | P58242 | RUVBL1 | 0.763 | 0.049164754 |
| Q99KI0 | ACO2 | 1.337 | 0.020549282 |  |  |  |  |
| Q3U7R1 | ESYT1 | 1.307 | 0.025087592 |  |  |  |  |

Proteins increased (top; green shading) or decreased (bottom; orange shading) >1.3 fold with p< 0.05 are shown.
