# Supplementary material for: The ATG5 interactome links clathrin-mediated vesicular trafficking with the autophagosome assembly machinery
Source: Autophagy Rep. 2022 Apr 7;1(1):88–118. doi: 10.1080/27694127.2022.2042054 (PMC9015699; doi:10.1080/27694127.2022.2042054)
Supplement: Supplemental Material [file KAUO_A_2042054_SM3247.zip › Supplementary information/Table S12.docx]

**Table S12.** Surface interactome changes upon starvation (ratio starved:fed state) in the different MEF rescue backgrounds.

| **WT GFP-ATG5 vs. GFP** | | | | | | | |
| --- | --- | --- | --- | --- | --- | --- | --- |
| ***Increased*** | | | | ***Decreased*** | | | |
| **Accession** | **Description** | **Starved/Fed mean** | ***P*-value** | **Accession** | **Description** | **Starved/Fed mean** | ***P*-value** |
| Q9R0P5 | DSTN | 1.775 | 0.020129119 | Q3TVX7 | SYPL | 0.499202051 | 0.021841626 |
| A2A813 | PARK7 | 1.617 | 0.044781611 | P11438 | LAMP1 | 0.579 | 0.004855501 |
| Q9R0B9 | PLOD2 | 1.473 | 0.025822975 | P04925 | PRNP | 0.664 | 0.046346883 |
| O09172 | GCLM | 1.323 | 0.027309868 |  |  |  |  |
|  | | | | | | | |
| **WT GFP-ATG5 vs. K130R GFP-ATG5** | | | | | | | |
| ***Increased*** | | | | ***Decreased*** | | | |
| **Accession** | **Description** | **Starved/Fed mean** | ***P*-value** | **Accession** | **Description** | **Starved/Fed mean** | ***P*-value** |
| Q9WVG6 | CARM1 | 1.707 | 0.027837244 | Q9EPR5 | SORCS2 | 0.377 | 0.033646884 |
| Q9CQ65 | MTAP | 1.590 | 0.038584358 | P53994 | RAB2A | 0.641 | 0.01405476 |
| E9PVA8 | GCN1 | 1.589 | 0.040604744 | Q05DV1 | POR | 0.695 | 0.023477359 |
| Q8CG48 | SMC2 | 1.537 | 0.036123316 |  |  |  |  |
| P26039 | TLN1 | 1.448 | 0.030744401 |  |  |  |  |
| Q9JKF1 | IQGAP1 | 1.379 | 0.037147102 |  |  |  |  |
|  | | | | | | | |
| **K130R GFP-ATG5 vs. GFP** | | | | | | | |
| ***Increased*** | | | | ***Decreased*** | | | |
| **Accession** | **Description** | **Starved/Fed mean** | ***P*-value** | **Accession** | **Description** | **Starved/Fed mean** | ***P*-value** |
| Q8BKE6 | CYP20A1 | 1.498 | 0.045389404 | P19096 | FASN | 0.687 | 0.039200995 |

Proteins increased (top; green shading) or decreased (bottom; orange shading) >1.3 fold with p< 0.05 are shown.
