# Supplementary material for: The ATG5 interactome links clathrin-mediated vesicular trafficking with the autophagosome assembly machinery
Source: Autophagy Rep. 2022 Apr 7;1(1):88–118. doi: 10.1080/27694127.2022.2042054 (PMC9015699; doi:10.1080/27694127.2022.2042054)
Supplement: Supplemental Material [file KAUO_A_2042054_SM3247.zip › Supplementary information/Table S2.docx]

**Table S2.** The wild-type GFP-ATG5 interactome.

| **Accession** | **DESCRIPTION** | **Unique peptides** | **WT:GFP** | **WT:K130R** | **K130R:GFP** | **Score** |
| --- | --- | --- | --- | --- | --- | --- |
| P60521 | GABARAPL2 | 6 | 100.000 | 100.000 | 0.010 | 71.66 |
| Q569L8 | CENPJ | 2 | 100.000 | 100.000 | 100.000 | 2.41 |
| Q3UV55 | NR1D1 | 2 | 100.000 | 100.000 | 0.040 | 0.00 |
| P39053 | DNM1 | 14 | 100.000 | 94.335 | 0.684 | 43.86 |
| O70405 | ULK1 | 9 | 100.000 | 39.869 | 100.000 | 13.22 |
| Q9JKY5 | HIP1R | 34 | 100.000 | 23.723 | 0.742 | 60.33 |
| Q3TRC8 | ARRB2 | 4 | 100.000 | 7.668 | 100.000 | 5.71 |
| Q8BW35 | Uncharacterized protein | 2 | 100.000 | 6.482 | 100.000 | 2.04 |
| Q8C0J2 | ATG16L1 | 13 | 100.000 | 6.478 | 27.903 | 342.83 |
| O70305 | ATXN2 | 2 | 100.000 | 5.058 | 100.000 | 1.85 |
| Q3TCY0 | RAB33B | 2 | 100.000 | 4.520 | 55.315 | 3.74 |
| D3YZ62 | MYO5A | 11 | 100.000 | 3.958 | 100.000 | 15.05 |
| Q8BTM5 | SLC2A1 | 2 | 100.000 | 3.945 | 100.000 | 0.00 |
| Q8VCC1 | HPGD | 7 | 100.000 | 3.372 | 26.545 | 32.91 |
| Q8R080 | GTSE1 | 4 | 100.000 | 3.092 | 77.471 | 5.69 |
| B1AWE0 | CLTA | 7 | 86.452 | 100.000 | 0.012 | 24.77 |
| Q9CPX6 | ATG3 | 18 | 81.825 | 100.000 | 0.010 | 324.34 |
| Q99J83 | ATG5 | 17 | 55.399 | 2.601 | 100.000 | 367.86 |
| F8VPL2 | PIK3C2A | 38 | 55.352 | 88.334 | 0.342 | 53.28 |
| F7AC41 | PUS7 | 2 | 55.194 | 2.549 | 40.840 | 0.00 |
| Q3TZU7 | SNX9 | 16 | 49.013 | 100.000 | 0.045 | 45.82 |
| Q8R3R8 | GABARAPL1 | 2 | 47.608 | 100.000 | 0.010 | 8.40 |
| Q68FD5 | CLTC | 74 | 46.149 | 100.000 | 0.114 | 600.82 |
| Q3TAB9 | ATG7 | 29 | 46.072 | 100.000 | 0.010 | 204.66 |
| Q3UIZ0 | GAK | 29 | 41.810 | 100.000 | 0.020 | 48.65 |
| Q8VD75 | HIP1 | 19 | 40.988 | 100.000 | 0.010 | 25.33 |
| G3X9G4 | DNM2 | 27 | 35.464 | 78.710 | 0.396 | 111.81 |
| Q9ESK9 | RB1CC1 | 39 | 31.434 | 100.000 | 0.011 | 48.78 |
| P62743 | AP2S1 | 3 | 28.313 | 7.944 | 2.200 | 7.62 |
| B7ZWC4 | IGF2R | 24 | 26.275 | 41.385 | 0.369 | 28.69 |
| P51863 | ATP6V0D1 | 2 | 26.249 | 2.909 | 11.648 | 4.51 |
| Q9D699 | GAS2 | 3 | 25.446 | 2.321 | 15.349 | 3.65 |
| Q9JKB1 | UCHL3 | 2 | 24.281 | 2.083 | 22.320 | 2.52 |
| G3UWG1 | GM10108 | 3 | 23.854 | 18.617 | 1.742 | 8.75 |
| Q6TXD4 | DNMBP | 8 | 23.310 | 100.000 | 0.010 | 5.19 |
| Q9DBW2 | INPP5B | 3 | 22.753 | 2.216 | 14.878 | 0.00 |
| Q99JB8 | PACSIN3 | 2 | 20.976 | 5.258 | 9.888 | 1.73 |
| Z4YJU8 | GOLGA2 | 4 | 20.770 | 2.116 | 25.105 | 1.88 |
| Q8K382 | DENND1A | 4 | 18.387 | 100.000 | 0.010 | 5.42 |
| Q9CQY1 | ATG12 | 6 | 18.097 | 100.000 | 0.010 | 48.36 |
| G3X956 | SUPT16 | 2 | 17.497 | 3.443 | 4.406 | 0.00 |
| D3Z656 | SYNJ1 | 13 | 17.100 | 38.822 | 0.615 | 10.62 |
| E9QAT4 | SEC16A | 8 | 17.048 | 4.347 | 3.953 | 9.04 |
| Q8CC03 | AP1G1 | 7 | 16.283 | 7.192 | 2.288 | 13.82 |
| P62192 | PSMC1 | 2 | 15.605 | 3.696 | 9.613 | 6.29 |
| P18828 | SDC1 | 3 | 15.263 | 4.064 | 0.491 | 2.08 |
| Q8BUK6 | HOOK3 | 4 | 14.959 | 3.502 | 100.000 | 7.53 |
| F6RAZ3 | ACADS | 2 | 13.991 | 14.156 | 1.000 | 5.43 |
| Q3TDG9 | STX12 | 2 | 13.990 | 100.000 | 0.088 | 0.00 |
| B2RQQ7 | CDC42BPB | 5 | 13.927 | 5.485 | 72.026 | 12.18 |
| Q3UP40 | PACSIN2 | 2 | 13.148 | 2.188 | 12.836 | 0.00 |
| E9QA74 | MYO18A | 3 | 12.559 | 10.277 | 1.222 | 1.84 |
| Q3TWV4 | AP2M1 | 14 | 12.296 | 7.057 | 1.099 | 34.76 |
| Q0PD42 | RAB13 | 2 | 11.818 | 4.300 | 2.749 | 6.11 |
| D3YZ06 | HSPB1 | 2 | 11.791 | 5.417 | 2.177 | 1.69 |
| Q9WUU7 | CTSZ | 4 | 11.683 | 2.934 | 7.891 | 6.97 |
| Q32ME1 | ATP2B4 | 2 | 11.243 | 100.000 | 0.010 | 1.82 |
| P17426 | AP2A1 | 21 | 10.909 | 15.395 | 0.609 | 83.06 |
| Q3TD51 | PICALM | 13 | 10.265 | 24.624 | 0.407 | 31.43 |
| D3Z2E3 | REPS1 | 4 | 9.675 | 47.542 | 0.273 | 3.92 |
| Q9DCE6 | DAB2 | 3 | 9.551 | 7.472 | 1.278 | 1.87 |
| P17427 | AP2A2 | 21 | 9.287 | 7.276 | 1.256 | 77.01 |
| P35585 | AP1M1 | 11 | 9.042 | 14.268 | 0.973 | 13.52 |
| Q5SVG5 | AP1B1 | 6 | 8.141 | 6.996 | 1.164 | 64.85 |
| Q9DBG3 | AP2B1 | 11 | 8.085 | 7.838 | 0.987 | 81.04 |
| Q3UHX2 | PDAP1 | 3 | 7.690 | 6.013 | 1.279 | 6.80 |
| Q6A009 | LTN1 | 3 | 7.541 | 2.226 | 3.904 | 3.31 |
| E9PYE2 | TYW3 | 2 | 7.280 | 2.288 | 3.182 | 2.38 |
| G3X920 | ARMC8 | 3 | 6.708 | 10.846 | 0.618 | 2.68 |
| D3Z7E5 | GSK3A | 4 | 6.089 | 2.657 | 2.037 | 2.33 |
| Q6PIU9 | FLJ45252 Homolog | 2 | 6.048 | 68.229 | 0.089 | 3.72 |
| Q920I9 | WDR7 | 2 | 5.877 | 6.022 | 0.463 | 1.93 |
| O54781 | SRPK2 | 4 | 5.847 | 4.766 | 0.583 | 8.29 |
| Q3V471 | LGALS3 | 2 | 5.752 | 3.893 | 1.477 | 1.78 |
| A2AUM2 | EIF2AK4 | 2 | 5.664 | 100.000 | 0.010 | 0.00 |
| Q80SW1 | AHCYL1 | 10 | 5.520 | 5.205 | 0.903 | 25.09 |
| H9KUZ8 | CEP41 | 2 | 5.499 | 2.720 | 2.021 | 1.89 |
| Q01320 | TOP2A | 3 | 5.434 | 2.332 | 2.330 | 1.97 |
| Q9DB05 | NAPA | 8 | 5.344 | 2.545 | 1.331 | 16.51 |
| P97390 | VPS45 | 4 | 5.330 | 2.621 | 23.458 | 3.58 |
| Q9CW46 | RAVER1 | 5 | 5.143 | 3.066 | 0.104 | 6.47 |
| A0A0A0MQN9 | TTC28 | 4 | 4.975 | 2.872 | 1.732 | 1.69 |
| Q3UAZ7 | HMGB2 | 2 | 4.951 | 3.456 | 1.433 | 3.78 |
| Q8BXV2 | BRI3BP | 2 | 4.937 | 3.257 | 1.516 | 3.38 |
| D3Z6W1 | MAP6 | 2 | 4.780 | 5.552 | 0.861 | 6.11 |
| D3Z7S0 | GET4 | 2 | 4.732 | 2.519 | 1.879 | 4.05 |
| Q9R061 | NUBP2 | 2 | 4.694 | 2.776 | 1.691 | 13.83 |
| Q8VHC3 | SELM | 2 | 4.661 | 2.910 |  | 2.11 |
| Q3TYL9 | CNP | 4 | 4.525 | 2.537 | 1.768 | 12.95 |
| O35682 | MYADM | 2 | 4.394 | 3.044 | 1.443 | 2.03 |
| Q80ZU1 | DCAF7 | 2 | 4.384 | 15.638 | 0.121 | 1.84 |
| P70271 | PDLIM4 | 5 | 4.363 | 3.587 | 1.085 | 5.58 |
| Q640N1 | AEBP1 | 2 | 4.325 | 3.305 | 1.309 | 1.61 |
| B7ZMQ9 | INPP4A | 2 | 4.317 | 3.778 | 0.857 | 1.72 |
| Q08091 | CNN1 | 8 | 4.278 | 7.478 | 0.551 | 29.74 |
| Q9EPK6 | SIL1 | 3 | 4.245 | 100.000 | 0.010 | 5.30 |
| Q9QYJ3 | DNAJB1 | 4 | 4.194 | 2.047 | 2.049 | 5.17 |
| Q3UHJ0 | AAK1 | 7 | 4.169 | 100.000 | 0.010 | 6.44 |
| A2AFQ9 | GEMIN5 | 8 | 4.099 | 2.086 | 2.406 | 8.94 |
| Q9QZA0 | CA5B | 2 | 4.068 | 11.774 | 0.284 | 0.00 |
| F8VQE9 | AGAP3 | 3 | 4.029 | 2.554 | 1.577 | 3.77 |
| P56389 | CDA | 2 | 3.948 | 3.280 | 1.204 | 2.89 |
| Q8C871 | SERPINB8 | 3 | 3.904 | 5.142 | 0.746 | 6.37 |
| F8WHM5 | GLG1 | 8 | 3.822 | 5.653 | 0.660 | 14.36 |
| Q6SLK2 | WNK1 | 4 | 3.796 | 3.984 | 0.953 | 0.00 |
| Q80XP8 | FAM76B | 2 | 3.716 | 2.580 | 1.440 | 4.02 |
| Q3UGK7 | WASL | 3 | 3.639 | 2.156 | 1.688 | 1.98 |
| F8VPN4 | AGL | 6 | 3.600 | 100.000 | 0.010 | 3.55 |
| Q3UXP1 | PHF6 | 2 | 3.471 | 2.400 | 1.446 | 0.00 |
| P62331 | ARF6 | 2 | 3.436 | 100.000 | 0.010 | 0.00 |
| Q8C078 | CAMKK2 | 4 | 3.368 | 14.148 | 0.086 | 1.71 |
| Q9CQV8 | YWHAB | 3 | 3.315 | 2.258 | 1.441 | 42.77 |
| Q08288 | LYAR | 3 | 3.292 | 2.756 | 1.194 | 1.70 |
| A0A0B4J1E5 | UCK2 | 2 | 3.289 | 11.609 | 0.088 | 5.75 |
| Q3TDW6 | APBB2 | 2 | 3.254 | 2.004 | 1.624 | 0.00 |
| F6XC25 | CC2D1B | 3 | 3.211 | 3.077 | 1.044 | 7.26 |
| Q6PAR5 | GAPVD1 | 2 | 3.208 | 4.789 | 0.670 | 0.00 |
| P80314 | CCT2 | 15 | 3.157 | 2.418 | 1.113 | 77.57 |
| Q80VY1 | HZGJ-LIKE | 2 | 3.108 | 6.041 | 0.893 | 6.05 |
| O08547 | SEC22B | 4 | 3.101 | 2.725 | 1.362 | 10.18 |
| Q8BTU6 | EIF4A2 | 4 | 3.062 | 22.427 | 0.082 | 45.68 |
| B1AZ15 | COBLL1 | 3 | 3.046 | 9.333 | 0.326 | 1.64 |
| Q61879 | MYH10 | 7 | 3.032 | 3.999 | 0.738 | 43.23 |
| Q69ZG0 | MKIAA1574 | 3 | 2.962 | 2.526 | 1.419 | 0.00 |
| Q0VF62 | BCAS3 | 4 | 2.941 | 3.530 | 1.144 | 1.60 |
| Q6P1G0 | HEATR6 | 3 | 2.923 | 2.417 | 1.051 | 1.86 |
| Q9WVS5 | CCTQ | 25 | 2.889 | 2.188 | 1.221 | 113.20 |
| Q60960 | KPNA1 | 3 | 2.870 | 2.013 | 1.807 | 39.65 |
| Q5JC28 | EPS15 | 4 | 2.861 | 100.000 | 0.010 | 1.63 |
| Q9D1M0 | SEC13 | 5 | 2.849 | 2.067 | 1.151 | 13.02 |
| Q8VDP2 | CXORF56 | 4 | 2.845 | 2.781 | 0.859 | 4.16 |
| D3YZU6 | HAGHL | 3 | 2.838 | 13.021 | 0.153 | 5.98 |
| Q3UFY8 | TRMT10C | 2 | 2.830 | 28.824 | 0.055 | 2.03 |
| P98192 | GNPAT | 3 | 2.801 | 12.088 | 0.197 | 3.49 |
| Q9JJ62 | TOP3B | 3 | 2.759 | 5.777 | 0.478 | 0.00 |
| G3X8U8 | PARG | 5 | 2.716 | 2.194 | 1.011 | 7.56 |
| Q9WUQ2 | PREB | 5 | 2.712 | 2.870 | 1.057 | 13.31 |
| Q6P9Q4 | FHOD1 | 5 | 2.705 | 2.896 | 0.696 | 7.81 |
| A2ALF0 | DNAJC8 | 3 | 2.701 | 3.215 | 0.840 | 6.57 |
| Q3TXV7 | HEXA | 2 | 2.693 | 2.561 | 1.052 | 0.00 |
| A1E2B8 | Inducible heat shock protein 70 | 10 | 2.683 | 2.243 | 1.226 | 89.40 |
| Q8BI72 | CDKN2AIP | 5 | 2.682 | 2.175 | 1.345 | 20.98 |
| Q3TUY5 | 5730455P16RIK | 3 | 2.680 | 2.366 | 1.133 | 3.77 |
| G5E896 | EDC4 | 11 | 2.680 | 2.640 | 0.845 | 15.38 |
| P27612 | PLAA | 9 | 2.672 | 2.632 | 0.534 | 11.59 |
| Q3U3U3 | GPC1 | 2 | 2.661 | 100.000 | 0.010 | 1.76 |
| E9QMH7 | IKBIP | 2 | 2.647 | 2.629 | 0.613 | 7.68 |
| Q9D6K8 | FUNDC2 | 2 | 2.644 | 2.536 | 1.042 | 0.00 |
| Q4VA53 | PDS5B | 2 | 2.633 | 100.000 | 0.010 | 4.23 |
| Q4V9X0 | PPP3CA | 2 | 2.624 | 100.000 | 0.010 | 2.30 |
| Q8BIJ7 | RUFY1 | 4 | 2.619 | 6.977 | 0.010 | 3.37 |
| E0CY23 | HSPA4L | 10 | 2.584 | 2.815 | 1.021 | 20.61 |
| A2BE92 | SET | 2 | 2.578 | 2.391 | 1.078 | 3.34 |
| A2A6U3 | SEPT9 | 11 | 2.570 | 2.013 | 1.393 | 22.47 |
| Q0KL02 | TRIO | 9 | 2.567 | 4.894 | 0.496 | 3.93 |
| D3Z3J6 | PAIP1 | 3 | 2.548 | 100.000 | 0.010 | 5.73 |
| Q9D289 | TRAPPC6B | 2 | 2.532 | 2.238 | 0.959 | 0.00 |
| Q8K297 | COLGALT1 | 7 | 2.514 | 2.002 | 1.310 | 9.60 |
| P80317 | CCT6A | 19 | 2.494 | 2.197 | 1.125 | 54.20 |
| E9QNY8 | SACS | 5 | 2.493 | 2.686 | 0.928 | 3.81 |
| Q99J95 | CDK9 | 8 | 2.490 | 2.384 | 1.337 | 35.68 |
| B2RWX4 | RINT1 | 2 | 2.475 | 3.410 | 0.726 | 0.00 |
| A2AWA9 | RABGAP1 | 2 | 2.473 | 3.809 | 0.649 | 6.03 |
| Q924H5 | RAD51C | 3 | 2.462 | 2.632 | 0.935 | 7.52 |
| D3Z198 | MRPS17 | 2 | 2.458 | 4.280 | 0.584 | 0.00 |
| H3BKG0 | CAV1 | 2 | 2.455 | 2.116 | 1.308 | 2.08 |
| Q3TX72 | FKBP5 | 2 | 2.445 | 3.308 | 0.739 | 4.11 |
| O89032 | SH3PXD2A | 2 | 2.443 | 14.036 | 0.010 | 4.57 |
| D3YU01 | PLEKHA1 | 3 | 2.431 | 2.002 | 11.216 | 3.39 |
| F6V084 | TMX1 | 2 | 2.425 | 2.475 | 0.623 | 13.55 |
| Q8C9Y7 | ECT2 | 2 | 2.422 | 2.495 | 0.951 | 0.00 |
| F6THK7 | IRAK1 | 2 | 2.419 | 2.107 | 1.148 | 7.49 |
| Q3TNH0 | TMPO | 4 | 2.418 | 2.306 | 0.981 | 2.04 |
| Q3TJ21 | PYCR2 | 6 | 2.394 | 2.872 | 1.301 | 18.20 |
| Q3TV90 | LUC7L | 2 | 2.368 | 4.244 | 0.329 | 8.87 |
| D3Z3F8 | SPG20 | 6 | 2.351 | 2.814 | 0.731 | 11.26 |
| Q8BI84 | MIA3 | 5 | 2.333 | 6.098 | 0.076 | 5.18 |
| K7Q751 | PTK2 | 6 | 2.330 | 2.087 | 0.897 | 10.55 |
| Q3TM37 | SNW1 | 2 | 2.319 | 2.791 | 0.831 | 3.81 |
| P02469 | LAMB1 | 5 | 2.311 | 2.192 | 0.632 | 13.56 |
| Q8VDM6 | HNRNPUL1 | 5 | 2.287 | 6.112 | 0.498 | 3.49 |
| Q3U896 | MTMR9 | 2 | 2.285 | 100.000 | 0.010 | 4.34 |
| Q8BVQ0 | WDR61 | 2 | 2.278 | 3.697 | 0.616 | 2.20 |
| Q8R216 | SIRT4 | 3 | 2.278 | 2.151 | 0.908 | 12.24 |
| P80315 | CCT4 | 17 | 2.271 | 2.062 | 1.016 | 106.00 |
| Q80ZM5 | H1FX | 3 | 2.263 | 2.464 | 1.021 | 12.98 |
| B2RY04 | DOCK5 | 2 | 2.255 | 2.625 | 0.859 | 4.01 |
| V9GXD1 | CCDC132 | 2 | 2.253 | 2.427 | 0.928 | 4.19 |
| Q8BK72 | MRPS27 | 3 | 2.239 | 2.655 | 0.843 | 7.70 |
| P63101 | YWHAZ | 8 | 2.232 | 2.298 | 0.883 | 53.22 |
| Q78HU3 | MVB12A | 3 | 2.223 | 2.978 | 0.812 | 5.42 |
| P35831 | PTPN12 | 4 | 2.206 | 2.942 | 0.749 | 5.78 |
| P11983 | TCP1 | 21 | 2.205 | 2.133 | 1.007 | 79.23 |
| A2AAY5 | SH3PXD2B | 2 | 2.188 | 2.364 | 0.926 | 2.12 |
| O08614 | UTRN | 16 | 2.174 | 3.039 | 0.618 | 29.15 |
| B2RY79 | DOCK9 | 10 | 2.157 | 26.680 | 0.050 | 11.63 |
| Q99LE7 | PXN | 2 | 2.146 | 2.963 | 0.652 | 1.63 |
| Q3TYJ0 | STUB1 | 2 | 2.143 | 4.205 | 0.631 | 6.22 |
| Q99PB4 | MAGED2 | 2 | 2.141 | 2.985 | 0.787 | 3.48 |
| Q6KAP1 | MFLJ00246 | 2 | 2.140 | 2.530 | 0.846 | 20.25 |
| P80316 | CCT5 | 19 | 2.138 | 2.273 | 0.928 | 51.65 |
| Q3TSX8 | TOMM70A | 5 | 2.131 | 5.047 | 0.413 | 11.94 |
| Q9CZP0 | UFSP1 | 2 | 2.106 | 2.282 | 0.923 | 0.00 |
| Q9D786 | HAUS5 | 2 | 2.101 | 2.311 | 0.904 | 4.11 |
| P80318 | CCT3 | 29 | 2.089 | 2.023 | 1.061 | 120.84 |
| P50544 | ACADVL | 18 | 2.075 | 2.100 | 0.841 | 66.17 |
| P69566 | RANBP9 | 2 | 2.060 | 2.370 | 0.869 | 2.06 |
| O70481 | UBR1 | 3 | 2.057 | 2.016 | 0.824 | 4.02 |
| D3Z030 | LRRC16A | 10 | 2.055 | 3.274 | 0.648 | 11.65 |
| Q8C650 | SEPT10 | 2 | 2.049 | 2.633 | 0.778 | 20.08 |
| Q3ULN6 | SCCPDH | 6 | 2.046 | 8.172 | 0.250 | 9.08 |
| Q9D0I4 | STX17 | 3 | 2.041 | 3.665 | 0.638 | 2.73 |
| Q3TNW7 | TBC1D1 | 2 | 2.033 | 3.118 | 0.652 | 3.46 |
| Q9JJV2 | PFN2 | 5 | 2.028 | 2.966 | 0.705 | 46.80 |
| A2AIW9 | PMPCA | 7 | 2.024 | 5.994 | 0.313 | 16.31 |
| E9Q3L2 | PI4KA | 4 | 2.022 | 2.966 | 1.448 | 1.98 |
| P70451 | FER | 3 | 2.013 | 100.000 | 0.010 | 5.79 |
| Q6ZPE2 | SBF1 | 6 | 2.013 | 2.728 | 1.496 | 5.65 |
| P51660 | HSD17B4 | 13 | 2.001 | 2.271 | 0.943 | 63.20 |

Putative interactors represented by 2 or more unique peptides ranked in order of: (i) wild type GFP-ATG5:GFP interactors (>2-fold enrichment); (ii) wild-type:mutant GFP-ATG5 interactors (>2-fold enrichment); (iii) score. Autophagy molecules are highlighted green; membrane trafficking molecules are highlighted yellow.
