# Supplementary material for: The ATG5 interactome links clathrin-mediated vesicular trafficking with the autophagosome assembly machinery
Source: Autophagy Rep. 2022 Apr 7;1(1):88–118. doi: 10.1080/27694127.2022.2042054 (PMC9015699; doi:10.1080/27694127.2022.2042054)
Supplement: Supplemental Material [file KAUO_A_2042054_SM3247.zip › Supplementary information/Table S3.docx]

**Table S3.** The K130R GFP-ATG5 interactome.

| **Accession** | **DESCRIPTION** | **Unique peptides** | **K130R:GFP** | **K130R:WT** | **WT:GFP** | **Score** |
| --- | --- | --- | --- | --- | --- | --- |
| Q9DC23 | DNAJC10 | 16 | 100.000 | 100.000 | 1.000 | 33.55 |
| Q61292 | LAMB2 | 9 | 100.000 | 100.000 | 1.000 | 14.88 |
| Q3UGP0 | DCLK1 | 5 | 100.000 | 100.000 | 1.000 | 11.07 |
| Q8CG19 | LTBP1 | 8 | 100.000 | 100.000 | 1.000 | 10.18 |
| Q3U5K8 | IFIT1 | 4 | 100.000 | 100.000 | 1.000 | 9.13 |
| Q9EQH2 | ERAP1 | 7 | 100.000 | 100.000 | 1.000 | 7.36 |
| Q3UUU0 | EMILIN1 | 6 | 100.000 | 100.000 | 1.000 | 6.73 |
| Q91VM5 | RBMXL1 | 3 | 100.000 | 100.000 | 1.000 | 6.00 |
| Q62356 | FSTL1 | 5 | 100.000 | 100.000 | 1.000 | 5.98 |
| Q8K173 | COL3A1 | 3 | 100.000 | 100.000 | 1.000 | 4.26 |
| D3Z1C5 | LDB1 | 2 | 100.000 | 100.000 | 1.000 | 3.71 |
| Q542W1 | IL1RN | 2 | 100.000 | 100.000 | 1.000 | 3.12 |
| Q9D787 | PPIL2 | 2 | 100.000 | 100.000 | 1.000 | 2.72 |
| F7C279 | BNIP1 | 2 | 100.000 | 100.000 | 1.000 | 2.58 |
| Q8VDC1 | FYCO1 | 2 | 100.000 | 100.000 | 1.000 | 2.57 |
| H7BWY5 | PARP9 | 2 | 100.000 | 100.000 | 1.000 | 2.17 |
| Q3UPI9 | POLE | 3 | 100.000 | 100.000 | 1.000 | 2.01 |
| E9Q7G0 | NUMA1 | 3 | 100.000 | 100.000 | 1.000 | 1.98 |
| F6Z1C2 | EFEMP2 | 2 | 100.000 | 100.000 | 1.000 | 1.83 |
| Q5NCU4 | SPARC | 2 | 100.000 | 100.000 | 1.000 | 1.74 |
| Q14AX3 | KCTD12 | 2 | 100.000 | 100.000 | 1.000 | 1.70 |
| P83870 | PHF5A | 3 | 100.000 | 100.000 | 1.000 | 1.62 |
| D3YUE0 | TREX1 | 3 | 100.000 | 22.219 | 1.000 | 7.52 |
| Q6AXC6 | DDX11 | 2 | 100.000 | 15.577 | 10.000 | 1.61 |
| Q9D6T0 | NOSIP | 3 | 100.000 | 14.439 | 1.000 | 2.14 |
| P11087 | COL1A1 | 11 | 100.000 | 14.097 | 1.949 | 26.73 |
| B2RXC8 | PPP2R3A | 2 | 100.000 | 13.596 | 1.000 | 1.69 |
| A0A087WS27 | FAM46A | 2 | 100.000 | 10.468 | 1.000 | 5.55 |
| P51949 | MNAT1 | 3 | 100.000 | 10.101 | 10.000 | 1.63 |
| B1B0C7 | HSPG2 | 8 | 100.000 | 2.538 | 1.000 | 1.65 |
| Q9CQW9 | IFITM3 | 2 | 77.242 | 77.242 | 1.000 | 1.68 |
| Q3TGL4 | FBLN2 | 19 | 76.850 | 17.559 | 1.341 | 53.74 |
| Q3UGQ1 | TRP53 | 10 | 75.272 | 37.923 | 1.000 | 15.36 |
| E9PX70 | COL12A1 | 24 | 72.752 | 11.089 | 1.645 | 30.54 |
| Q9Z315 | SART1 | 2 | 72.471 | 72.471 | 2.059 | 1.82 |
| Q05BJ7 | OAS1G | 2 | 42.294 | 32.177 | 1.314 | 4.71 |
| Q8BG46 | ANKRD10 | 2 | 35.353 | 16.644 | 2.124 | 2.86 |
| Q9JHT5 | AMMECR1 | 3 | 34.905 | 3.667 | 15.534 | 6.58 |
| Q922M3 | KCTD10 | 2 | 33.480 | 33.480 | 0.830 | 3.66 |
| O08807 | PRDX4 | 4 | 28.253 | 4.080 | 1.326 | 59.69 |
| Z4YJU8 | GOLGA2 | 2 | 25.105 | 2.116 | 20.770 | 1.88 |
| P09055 | ITGB1 | 6 | 24.298 | 5.649 | 3.946 | 6.13 |
| Q3TAP5 | TRA2A | 2 | 22.381 | 14.767 | 1.516 | 2.06 |
| A0A087WPL1 | PDLIM2 | 2 | 22.370 | 14.826 | 1.509 | 4.53 |
| Q91ZX7 | LRP1 | 38 | 22.261 | 5.078 | 2.229 | 56.74 |
| Q9WVH9 | FBLN5 | 2 | 18.774 | 16.175 | 0.010 | 2.24 |
| Q8WTY4 | CIAPIN1 | 6 | 17.542 | 5.418 | 2.429 | 16.29 |
| Q3UY34 | C12orf43 | 2 | 16.114 | 7.697 | 2.094 | 5.58 |
| Q5FWI3 | TMEM2 | 4 | 14.656 | 12.720 | 1.152 | 4.01 |
| B7ZWI2 | 1110005A23RIK | 4 | 14.367 | 6.662 | 1.800 | 6.85 |
| P07742 | RRM1 | 13 | 14.111 | 9.287 | 1.162 | 27.98 |
| Q9CT37 | HNRNPR | 2 | 13.079 | 10.063 | 1.300 | 15.09 |
| Q80U95 | UBE3C | 3 | 12.107 | 7.684 | 1.576 | 4.27 |
| Q8BJY1 | PSMD5 | 2 | 12.032 | 9.537 | 1.262 | 1.79 |
| A2RSY6 | TRMT1L | 2 | 11.434 | 11.624 | 0.984 | 1.65 |
| Q6ZQJ9 | NCAPH | 5 | 11.415 | 8.735 | 1.708 | 6.92 |
| A2A9P2 | NADK | 3 | 11.253 | 2.019 | 6.119 | 6.65 |
| Q3V028 | CYLD | 2 | 11.138 | 7.927 | 1.405 | 1.78 |
| Q78E06 | EIF2AK2 | 2 | 10.817 | 12.948 | 0.835 | 4.37 |
| Q921Q3 | ALG1 | 2 | 10.253 | 8.969 | 1.143 | 1.92 |
| P04184 | TK1 | 4 | 10.173 | 2.777 | 5.029 | 4.64 |
| Q61398 | PCOLCE | 8 | 9.728 | 3.787 | 1.688 | 18.09 |
| B9EKC1 | PASK | 4 | 9.400 | 7.213 | 1.811 | 1.60 |
| P18406 | CYR61 | 6 | 8.731 | 8.030 | 2.033 | 8.10 |
| Q8VHC5 | CABP4 | 2 | 8.547 | 5.743 | 1.488 | 2.13 |
| A2AKI5 | ITGAV | 2 | 7.607 | 3.034 | 2.507 | 1.96 |
| Q3TDD1 | LDLR | 6 | 6.896 | 4.024 | 1.762 | 9.26 |
| Q6GU23 | STAT3 | 17 | 6.773 | 2.691 | 1.591 | 26.08 |
| Q9R1P1 | PSMB3 | 3 | 6.612 | 5.801 | 1.317 | 3.52 |
| Q3TC83 | NLE1 | 3 | 6.456 | 2.828 | 2.283 | 1.86 |
| P24288 | BCAT1 | 7 | 6.424 | 2.326 | 1.777 | 15.04 |
| F8WIE5 | HECTD1 | 2 | 6.239 | 15.351 | 0.010 | 1.64 |
| P48759 | PTX3 | 7 | 5.720 | 6.171 | 1.000 | 11.94 |
| Q99J10 | CTU1 | 4 | 5.674 | 13.305 | 0.141 | 6.21 |
| G3UXB4 | CTU2 | 5 | 5.666 | 3.440 | 1.949 | 18.45 |
| E9Q197 | GLOD4 | 9 | 5.513 | 1.972 | 1.965 | 32.82 |
| Q3U8R9 | TXNL1 | 8 | 5.453 | 2.482 | 2.580 | 25.44 |
| Q9WVQ5 | APIP | 7 | 5.316 | 2.323 | 1.229 | 18.86 |
| F6WM75 | CCDC167 | 2 | 5.051 | 2.023 | 2.497 | 4.00 |
| Q8VBZ3 | CLPTM1 | 2 | 4.678 | 8.796 | 0.532 | 4.16 |
| Q3THQ5 | STIP1 | 11 | 4.565 | 2.055 | 2.190 | 19.95 |
| G3UYB1 | CHEK1 | 5 | 4.492 | 3.640 | 1.155 | 12.03 |
| Q7TMW1 | RANGAP1 | 13 | 4.439 | 2.355 | 1.507 | 27.07 |
| B2RUG7 | ZFR | 5 | 4.218 | 11.632 | 1.766 | 5.14 |
| P70302 | STIM1 | 2 | 4.212 | 3.387 | 1.244 | 1.61 |
| Q8C050 | RPS6KA5 | 2 | 4.199 | 2.645 | 1.588 | 1.62 |
| E9Q7B0 | P4HA1 | 5 | 3.830 | 4.598 | 0.936 | 6.62 |
| Q8BH83 | ANKRD9 | 6 | 3.643 | 2.552 | 1.193 | 6.40 |
| Q3U4J9 | CDK6 | 8 | 3.604 | 2.140 | 1.525 | 48.71 |
| Q3UKJ7 | SMU1 | 3 | 3.460 | 2.223 | 1.557 | 5.68 |
| P30285 | CDK4 | 8 | 3.331 | 2.325 | 1.205 | 49.91 |
| A2AU61 | RALY | 4 | 3.282 | 2.019 | 1.916 | 6.97 |
| Q8C872 | TFRC | 5 | 3.139 | 3.098 | 1.077 | 10.53 |
| Q8BGR9 | UBLCP1 | 2 | 3.122 | 2.246 | 1.627 | 1.72 |
| B1AQR8 | LGALS9 | 5 | 2.479 | 8.303 | 0.497 | 22.60 |
| G3X8P6 | TXNRD3 | 5 | 2.414 | 2.137 | 0.140 | 10.77 |
| Q9JMH6 | TXNRD1 | 7 | 2.407 | 2.927 | 0.998 | 17.86 |
| B2RQ68 | LUZP1 | 3 | 2.389 | 11.894 | 1.300 | 7.79 |
| Q9R1S8 | CAPN7 | 3 | 2.302 | 1.992 | 1.254 | 1.90 |
| Q8BHK9 | ERCC6L | 3 | 2.277 | 2.413 | 1.947 | 3.63 |
| E9Q555 | RNF213 | 59 | 2.235 | 2.579 | 0.941 | 80.39 |
| Q6PE06 | KLHDC4 | 4 | 2.176 | 2.884 | 0.581 | 4.43 |

Putative interactors represented by 2 or more unique peptides ranked in order of: (i) K130R GFP-ATG5:GFP interactors (>2-fold enrichment); (ii) K130R GFP-ATG5:WT GFP-ATG5 interactors (>2-fold enrichment); (iii) score. Autophagy molecules are highlighted green; membrane trafficking molecules are highlighted yellow.
