# Supplementary material for: The ATG5 interactome links clathrin-mediated vesicular trafficking with the autophagosome assembly machinery
Source: Autophagy Rep. 2022 Apr 7;1(1):88–118. doi: 10.1080/27694127.2022.2042054 (PMC9015699; doi:10.1080/27694127.2022.2042054)
Supplement: Supplemental Material [file KAUO_A_2042054_SM3247.zip › Supplementary information/Table S5.docx]

**Table S5.** The GFP-ATG5 interactome in the *atg3* null background.

| **Accession** | **Description** | **Unique peptides** | **WT:GFP** | **WT:K130R** | **K130R:GFP** | **Score** |
| --- | --- | --- | --- | --- | --- | --- |
| Q3TDQ5 | ATG16L1 | 31 | 100.000 | 6.120 | 32.964 | 432.04 |
| Q6KAU8 | ATG16L2 | 18 | 100.000 | 3.292 | 100.000 | 156.16 |
| Q9CQY1 | ATG12 | 5 | 44.585 | 15.343 | 2.043 | 68.65 |
| Q9CPX6 | ATG3 | 16 | 43.193 | 100.000 | 0.010 | 351.60 |
| P60521 | GABARAPL2 | 6 | 38.693 | 100.000 | 0.010 | 200.07 |
| Q80VP0 | TECPR1 | 37 | 26.344 | 0.975 | 40.957 | 519.22 |
| Q3TAB9 | ATG7 | 22 | 22.541 | 100.000 | 0.152 | 122.13 |
| Q8BJL1 | FBXO30 | 2 | 19.013 | 42.700 | 0.320 | 2.26 |
| Q99J83 | ATG5 | 18 | 18.431 | 0.640 | 73.999 | 690.24 |
| P62192 | PSMC1 | 3 | 11.558 | 1.838 | 6.287 | 14.06 |
| Q9DAX9 | APPBP2 | 3 | 7.636 | 0.371 | 20.567 | 8.94 |
| Q68FD5 | CLTC | 50 | 7.517 | 11.637 | 0.665 | 310.55 |
| Q9QZZ6 | DPT | 3 | 5.980 | 0.294 | 12.435 | 19.01 |
| E9Q9A9 | OAS2 | 20 | 5.926 | 0.530 | 100.000 | 55.25 |
| Q3UIZ0 | GAK | 12 | 5.614 | 25.220 | 0.405 | 27.14 |
| Q03350 | THBS2 | 5 | 4.939 | 0.530 | 67.590 | 20.45 |
| Q3TJ69 | SERPINB9B | 6 | 4.919 | 1.652 | 1.645 | 12.80 |
| P39053 | DNM1 | 2 | 4.848 | 3.489 | 3.567 | 30.78 |
| B1AQR8 | LGALS9 | 4 | 4.761 | 3.392 | 1.187 | 6.31 |
| E9QP46 | SYNE2 | 2 | 4.325 | 100.000 | 0.010 | 4.39 |
| B2RXT5 | GPI1 | 3 | 4.047 | 0.835 | 5.621 | 4.25 |
| B1AWE0 | CLTA | 2 | 4.040 | 8.772 | 0.461 | 4.04 |
| P61804 | DAD1 | 3 | 3.952 | 0.922 | 0.788 | 18.58 |
| F8VPL2 | PI3KC2A | 10 | 3.836 | 11.818 | 0.300 | 19.49 |
| Q3TCY0 | RAB33B | 4 | 3.736 | 5.842 | 0.628 | 10.01 |
| P10649 | GSTM1 | 2 | 3.368 | 1.214 | 2.775 | 3.97 |
| P62814 | ATP6V1B2 | 3 | 3.293 | 1.494 | 1.875 | 9.26 |
| O35646 | CAPN6 | 8 | 2.952 | 0.909 | 3.153 | 22.75 |
| A2RSJ4 | UHRF1BP1L | 2 | 2.906 | 91.980 | 0.032 | 4.14 |
| B9EJ77 | TANC1 | 4 | 2.902 | 2.167 | 1.398 | 9.96 |
| P63154 | CRNKL1 | 2 | 2.887 | 11.270 | 0.083 | 5.09 |
| Q9CZK0 | SNX9 | 7 | 2.599 | 3.712 | 0.687 | 20.79 |
| P41731 | CD63 | 2 | 2.578 | 2.932 | 1.000 | 1.81 |
| Q3U1H7 | SNX18 | 2 | 2.555 | 3.208 | 0.796 | 1.62 |
| Q5NCB5 | IRGM1 | 3 | 2.553 | 0.761 | 4.725 | 11.45 |
| Q3TRC8 | ARRB2 | 2 | 2.549 | 2.637 | 0.967 | 1.88 |
| M0QW74 | MTMR3 | 2 | 2.521 | 100.000 | 0.013 | 6.81 |
| Q3T9X3 | DNM2 | 8 | 2.500 | 2.710 | 0.917 | 48.27 |
| B2RRF0 | PTPRK | 3 | 2.427 | 0.741 | 44.851 | 7.89 |
| Q60575 | KIF1B | 3 | 2.381 | 1.181 | 3.083 | 22.94 |
| Q9DC42 | CD97 | 5 | 2.372 | 2.666 | 0.794 | 11.63 |
| Q921W0 | CHMP1A | 2 | 2.336 | 0.817 | 2.859 | 6.79 |
| Q8BIF7 | GRWD1 | 2 | 2.316 | 17.317 | 0.086 | 3.90 |
| Q80UP1 | HIP1R | 3 | 2.311 | 81.993 | 0.028 | 3.35 |
| Q9D154 | SERPINB1A | 4 | 2.262 | 0.618 | 7.558 | 5.00 |
| Q69ZA7 | MKIAA1769 | 2 | 2.248 | 1.473 | 1.526 | 7.98 |
| O08797 | SERPINB9 | 4 | 2.202 | 0.696 | 2.814 | 20.40 |
| Q9QYR6 | MAP1A | 3 | 2.157 | 1.281 | 1.341 | 10.87 |
| Q61749 | EIF2B4 | 3 | 2.130 | 2.184 | 1.115 | 14.16 |
| Q9CQV8 | YWHAB | 5 | 2.114 | 1.804 | 1.142 | 113.95 |
| E9Q555 | RNF213 | 113 | 2.110 | 1.379 | 1.568 | 527.03 |
| Q9WTQ5 | AKAP12 | 13 | 2.086 | 2.930 | 0.606 | 28.85 |
| B2RQS1 | STRN3 | 3 | 2.074 | 1.089 | 1.904 | 4.44 |
| Q5MJ56 | MYO7A | 2 | 2.062 | 2.789 | 0.631 | 4.27 |
| Q9DB90 | SMG9 | 2 | 2.044 | 3.343 | 0.586 | 4.39 |
| Q8K234 | OASL2 | 7 | 2.034 | 0.499 | 3.447 | 31.04 |
| Q3TW11 | STAT1 | 25 | 2.030 | 0.962 | 2.197 | 138.03 |
| P11928 | OAS1A | 4 | 2.018 | 0.638 | 2.664 | 13.89 |
| Q9ER88 | DAP3 | 2 | 2.005 | 1.533 | 1.484 | 3.36 |

Putative interactors represented by 2 or more unique peptides ranked in order of: (i) wild type GFP-ATG5:GFP interactors (>2-fold enrichment); (ii) wild-type:mutant GFP-ATG5 interactors (>2-fold enrichment); (iii) score. Autophagy molecules are highlighted green; membrane trafficking molecules are highlighted yellow.
