# Supplementary material for: The ATG5 interactome links clathrin-mediated vesicular trafficking with the autophagosome assembly machinery
Source: Autophagy Rep. 2022 Apr 7;1(1):88–118. doi: 10.1080/27694127.2022.2042054 (PMC9015699; doi:10.1080/27694127.2022.2042054)
Supplement: Supplemental Material [file KAUO_A_2042054_SM3247.zip › Supplementary information/Table S7.docx]

**Table S7.** Surface interactome in WT GFP-ATG5 vs. GFP in the fed state.

| ***Increased*** | | | | ***Decreased*** | | | |
| --- | --- | --- | --- | --- | --- | --- | --- |
| **Accession** | **Description** | **Mean**  **WT : GFP** | ***P*-value** | **Accession** | **Description** | **Mean**  **WT : GFP** | ***P*-value** |
| P52800 | EFNB2 | 2.730 | 0.035033748 | P70202 | LXN | 0.564 | 0.030098094 |
| Q9JKF6 | PVRL1 | 2.316 | 0.010530861 | Q9R0P5 | DSTN | 0.595 | 0.017489458 |
| A2A8L5 | PTPRF PE | 2.205 | 0.022140881 | A0A067XG53 | CASK | 0.597 | 0.015246012 |
| Q9QXX0 | JAG1 | 2.044 | 0.002498732 | Q05DU8 | RRM1 | 0.605 | 0.028377966 |
| Q3UXH8 | HDAC2 | 1.765 | 0.018127927 | Z4YL78 | CKAP5 | 0.616 | 0.018539603 |
| Q61876 | Uncharacterized protein | 1.649 | 0.034883794 | Q9QYF9 | NDRG3 | 0.634 | 0.02342693 |
| Q3TDB6 | SLC31A1 | 1.533 | 0.034144225 | Q6QHF0 | TNFRSF1A | 0.640 | 0.022715216 |
| Q9CSH0 | HNRNPLL | 1.445 | 0.005323671 | Q91VE0 | SLC27A4 | 0.641 | 0.032681628 |
| Q3TDN0 | DISP1 | 1.434 | 0.018178448 | F8VQD7 | PTPRG | 0.642 | 0.017172075 |
| Q3TJG5 | SLC12A4 | 1.428 | 0.002214814 | Q64337 | SQSTM1 | 0.662 | 0.045255171 |
| Q6PB66 | LRPPRC | 1.421 | 0.022707129 | P27046 | MAN2A1 | 0.664 | 0.044194909 |
| P62814 | ATP6V1B2 | 1.420 | 0.0041942 | Q3UDS4 | SQRDL | 0.699 | 0.016460184 |
| Q3U449 | BPNT1 | 1.379 | 0.032157651 | Q9DBT5 | AMPD2 | 0.731 | 0.03829524 |
| Q8BFY6 | PEF1 | 1.323 | 0.012362173 | P70302 | STIM1 | 0.755 | 0.042594089 |
| A0A0A0U6W1 | Envelope protein | 1.317 | 0.044056924 | P47738 | ALDH2 | 0.758 | 0.041949934 |
|  |  |  |  | O09172 | GCLM | 0.762 | 0.044379611 |

These represent the proteins whose expression is increased (green shading) or decreased (orange shading) >1.3 fold with p< 0.05. These data are depicted diagrammatically in **Fig. 5B**.
