# Supplementary material for: The ATG5 interactome links clathrin-mediated vesicular trafficking with the autophagosome assembly machinery
Source: Autophagy Rep. 2022 Apr 7;1(1):88–118. doi: 10.1080/27694127.2022.2042054 (PMC9015699; doi:10.1080/27694127.2022.2042054)
Supplement: Supplemental Material [file KAUO_A_2042054_SM3247.zip › Supplementary information/Table S8.docx]

**Table S8.** Surface interactome in WT GFP-ATG5 vs. K130R GFP in the fed state.

| ***Increased*** | | | | ***Decreased*** | | | |
| --- | --- | --- | --- | --- | --- | --- | --- |
| **Accession** | **Description** | **Mean**  **WT : K130R** | ***P*-value** | **Accession** | **Description** | **Mean**  **WT : K130R** | ***P*-value** |
| E9PVD3 | DCHS1 | 2.334 | 0.0016836 | E9PV48 | IFIT3B | 0.013 | 0.00094788 |
| P98156 | VLDLR | 2.063 | 0.04272076 | F6VQ81 | TPD52L2 | 0.551 | 0.03955201 |
| P70207 | PLXNA2 | 1.917 | 0.03147214 | Q91VE0 | SLC27A4 | 0.600 | 0.03470227 |
| Q8BP67 | RPL24 | 1.759 | 0.01573821 | Q62087 | PON3 | 0.607 | 0.00355953 |
| O35607 | BMPR2 | 1.739 | 0.02682914 | P27046 | MAN2A1 | 0.620 | 0.01859173 |
| A2ATK9 | FAM171A1 | 1.718 | 0.02862772 | G3UXZ5 | PSME1 | 0.645 | 0.01229071 |
| P62754 | RPS6 | 1.708 | 0.00477564 | Q9CQ43 | DUT | 0.648 | 0.04658237 |
| Q91VD8 | PCDHB17 | 1.680 | 8.3049E-05 | Q01730 | RSU1 | 0.653 | 0.03962807 |
| E9QM38 | SLC12A2 | 1.663 | 0.02232769 | P45952 | ACADM | 0.662 | 0.01518668 |
| Q9D6G1 | HNRNPAB | 1.621 | 0.00407523 | P70302 | STIM1 | 0.672 | 0.0484652 |
| O89051 | ITM2B | 1.620 | 0.04344145 | O78207 | H2-D1 | 0.674 | 0.01256854 |
| Q99KP6 | PRPF19 | 1.619 | 0.0285495 | P26043 | RDX | 0.685 | 0.03325535 |
| Q7TT36 | ADGRA3 | 1.574 | 0.02674282 | P70303 | CTPS2 | 0.689 | 0.04017958 |
| Q60737 | CSNK2A1 | 1.566 | 0.00374756 | Q8BJY1 | PSMD5 | 0.702 | 0.03550639 |
| Q8VIK5 | PEAR1 | 1.552 | 0.02106129 | P97429 | ANXA4 | 0.704 | 0.01723249 |
| A2A699 | FAM171A2 | 1.538 | 0.04764387 | O88587 | COMT | 0.705 | 0.03218573 |
| E9PUQ9 | PIEZO1 | 1.450 | 0.02592485 | Q9D071 | MMS19 | 0.7076 | 0.01865301 |
| Q3U561 | RPL10A | 1.494 | 0.0203584 | A0A0R4J0H7 | NCAPD2 | 0.709 | 0.01514302 |
| Q9D074 | MGRN1 | 1.485 | 0.01024261 | E9Q855 | SCAMP3 | 0.712 | 0.0137413 |
| Q8C0I1 | AGPS | 1.458 | 0.04866581 | F8VQC1 | SRP72 | 0.727 | 0.04605419 |
| Q3TF81 | RPP30 | 1.437 | 0.02715007 | P31938 | MAP2K1 | 0.733 | 0.00360613 |
| Q61090 | FZD7 | 1.396 | 0.01208308 | P26039 | TLN1 | 0.734 | 0.04548505 |
| G3UYZ1 | IGSF8 | 1.361 | 0.00133379 | O35685 | NUDC | 0.747 | 0.0295591 |
| Q9WV91 | PTGFRN | 1.342 | 0.01585211 | Q99JW7 | CDK1 | 0.754 | 0.02838882 |
| P97792 | CXADR | 1.330 | 0.03203783 |  |  |  |  |
| Q8BKG3 | PTK7 | 1.330 | 0.01252472 |  |  |  |  |
| E9PXY1 | CUL4B | 1.322 | 0.01816524 |  |  |  |  |
| P82347 | SGCD | 1.317 | 0.04105339 |  |  |  |  |

These represent the proteins whose expression is increased (green shading) or decreased (orange shading) >1.3 fold with p< 0.05. These data are depicted diagrammatically in **Fig. 5C**.
