# Supplementary material for: The ATG5 interactome links clathrin-mediated vesicular trafficking with the autophagosome assembly machinery
Source: Autophagy Rep. 2022 Apr 7;1(1):88–118. doi: 10.1080/27694127.2022.2042054 (PMC9015699; doi:10.1080/27694127.2022.2042054)
Supplement: Supplemental Material [file KAUO_A_2042054_SM3247.zip › Supplementary information/Table S9.docx]

**Table S9.** Surface interactome in K130R GFP vs GFP in the fed state.

| ***Increased*** | | | | ***Decreased*** | | | |
| --- | --- | --- | --- | --- | --- | --- | --- |
| **Accession** | **Description** | **Mean**  **K130R : GFP** | ***P*-value** | **Accession** | **Description** | **Mean**  **K130R : GFP** | ***P*-value** |
| Q9JHJ8 | ICOSLG | 3.611 | 0.031519 | P57787 | SLC16A3 | 0.439 | 0.0299909 |
| B2RWZ0 | ABI3BP | 2.341 | 0.020331 | P02469 | LAMB1 | 0.516 | 0.0196555 |
| Q9CQ62 | DECR1 | 1.879 | 0.0399712 | Q3TJH1 | GNAI3 | 0.597 | 0.0474658 |
| Q99J39 | MLYCD | 1.813 | 0.0099304 | Q6PDG0 | NUP205 | 0.599 | 0.0424557 |
| Q3UAG2 | PGD | 1.634 | 0.0203635 | P40240 | CD9 | 0.599 | 0.0345749 |
| P08030 | APRT | 1.566 | 0.0261245 | A0A0R4J097 | TGFBR3 | 0.605 | 0.0305692 |
| Q9QXB9 | DRG2 | 1.560 | 0.0402071 | Q8R373 | CLMP | 0.649 | 0.0066052 |
| Q62186 | SSR4 | 1.444 | 0.0066468 | Q8BLU0 | FLRT2 | 0.656 | 0.0163612 |
| Q8BGX2 | C19ORF52 | 1.435 | 0.023599 | F8VQJ3 | LAMC1 | 0.668 | 0.0400735 |
| Q8C5P5 | NT5DC1 | 1.4172 | 0.015404 | Q8BUM1 | TARDBP | 0.682 | 0.0066638 |
| Q9CQ43 | DUT | 1.338 | 0.0447269 | F8VQD7 | PTPRG | 0.689 | 0.0383892 |
| P35700 | PRDX1 | 1.330 | 0.028688 | Q9QYF9 | NDRG3 | 0.699 | 0.0481767 |
| Q3U8R9 | TXNL1 | 1.328 | 0.0287701 | Q9DBV4 | MXRA8 | 0.701 | 0.0287062 |
| A0A0U1RNT6 | AUH | 1.305 | 0.048153 | A2ATK9 | FAM171A1 | 0.702 | 0.038105 |
|  |  |  |  | Q3TCZ2 | SLC29A1 | 0.708 | 0.0211726 |
|  |  |  |  | P70424 | ERBB2 | 0.711 | 0.0337593 |
|  |  |  |  | O35188 | CX3CL1 | 0.721 | 0.0369577 |
|  |  |  |  | Q61090 | FZD7 | 0.725 | 0.0225818 |
|  |  |  |  | A0A0R4J0A9 | LRP6 | 0.751 | 0.0082895 |
|  |  |  |  | Q8BKG3 | PTK7 | 0.755 | 0.0053365 |
|  |  |  |  | P97351 | RPS3A | 0.761 | 0.0309467 |

These represent the proteins whose expression is increased (green shading) or decreased (orange shading) >1.3 fold with p< 0.05. These data are depicted diagrammatically in **Fig. 5D**.
